# Design and Molecular dynamic Investigations of 7,8-Dihydroxyflavone Derivatives as Potential Neuroprotective Agents Against Alpha-synuclein

**DOI:** 10.1038/s41598-020-57417-9

**Published:** 2020-01-17

**Authors:** Mohankumar Thangavel, Vivek Chandramohan, Lalithamba Haralur Shankaraiah, Richard L. Jayaraj, Kumaradhas Poomani, Sivanandam Magudeeswaran, Hunday Govindasamy, Rajendran Vijayakumar, Balakrishnan Rangasamy, Manimaran Dharmar, Elangovan Namasivayam

**Affiliations:** 1 Department of Biotechnology, School of Biosciences, Periyar University, Salem, 636011 Tamilnadu India; 2 Department of Biotechnology, Siddaganga Institute of Technology, Tumakuru, 572103 Karnataka India; 3 Department of Chemistry, Siddaganga Institute of Technology, Tumakuru, 572103 Karnataka India; 4 Department of Pharmacology and Therapeutics, College of Medicine and Health Sciences, Al-Ain, Abudhabi 17666 United Arab Emirates; 5 Department of Physics, School of Physical Sciences, Periyar University, Salem, 636011 Tamilnadu India; 6 Department of Biology, College of Science in Zulfi, Majmaah University, Majmaah, 11952 Saudi Arabia

**Keywords:** Virtual drug screening, Molecular dynamics

## Abstract

Parkinson’s disease (PD) is the second most common neurodegenerative disorder caused due to loss of dopaminergic neurons in substantia nigra pars compacta, which occurs the presence of Lewy bodies made up of Alpha-synuclein (ASN) aggregation resulting in neuronal death. This study aims to identify potent 7,8-Dihydroxyflavone (DHF) derivatives to inhibit the ASN aggregation from *in silico* analysis. Molecular docking study reveals that carbamic ester derivatives of DHF [DHF-BAHPC (**8q**), DHF-BAHPEC (**8s**), DHF-BAHEC (**8p**), DHF-BDOPC (**8c**), DHF-BAPEC (**8n**) and DHF-BAMC (**8h**)] have good binding affinity towards ASN, when compared with DHF and L-DOPA; their docking score values are −16.3120, −16.1875, −15.2223, −14.3118, −14.2893, –14.2810, −14.0383, and −9.1560 kcal/mol respectively. The *in silico* pharmacological evaluation shows that these molecules exhibit the drug-likeness and ADMET properties. Molecular dynamics simulation confirms the stability of the molecules with ASN. The intermolecular interaction analyzed under the dynamic condition, allows to identify the candidate which potentially inhibits ASN aggregation. Hence, we propose that DHF derivatives are the potential lead drug molecules and preclinical studies are needed to confirm the promising therapeutic ability against PD.

## Introduction

Parkinson’s disease (PD) is a common neurodegenerative disorder characterized by impairment of motor functions due to complete loss of dopamine in the midbrain. Clinically, PD characterized by behavioral impairments such as rigidity, tremor, postural instability and bradykinesia^1^. Alpha-synuclein (ASN), a PD associated protein, misfolds and accumulates in the brain through protein aggregation. Post-mortem studies showed that these aggregated filamentous termed as a Lewy body, the neurotoxicity is a main factor involved in PD and other disorders such as Alzheimer and Prion disease^2^. Studies report that the loss of dopaminergic neurons in PD is partly due to the overexpression of ASN in the cytoplasm of neurons. ASN expression increases in the substantia nigra with the response of age in rhesus monkeys and humans^3^. The unfolded ASN protein does not have a secondary structure under the physiological condition. However, changes in various environmental factors (agitation, ion strength, pH) induces the formation of amyloid-like fibrils and ASN aggregates *in vitro*^4^. Comparatively, ASN has more cytotoxicity than the amyloid proteins, which generate amyloid-like fibrils^5^. To prevent the aggregation of protein and resultant proteotoxicity, therapeutic medicines could provide a significant neuroprotective effect against ASN aggregation^6,7^. However, these therapeutic medicines to inhibit the amyloid fibril formation, especially L-DOPA enhance the anti-cytotoxicity^8^. Bodner *et al*. reported that B2 (5-[4-(4-chlorobenzoyl)-1-piperazinyl]-8-nitro-quinoline) compound accelerates inclusion formation in PD and Huntington’s disease^9^. The plant extracts and phytoconstituents targeting ASN aggregation, oligomerization and fibrillation to reduce ASN toxicity in PD model^10^. The *in vitro* analysis states that the hydroxyl group moiety and nitrogen containing groups are important to inhibit the aggregation of ASN in different stages (oligomerization, fibrillation and aggregation). In addition, the binding of phytochemical molecules stabilizes the intrinsic structure of ASN. Similarly, in the present study, the DHF derivatives have hydroxyl and amine group; it may prevent the aggregation of ASN. Also, the monomer is the primary stage that leads to developing end stages of oligomeric and fibrils^11,12^, but the ASN aggregation is a major component of Lewy bodies. Furthermore, the folding of a monomer is very low compared with dimer and oligomeric forms. However, the inhibition of monomer prevents the aggregation of ASN. Numerous laboratory reports have been published in recent years concerning ASN related mechanisms that may be responsible for the observed neurodegeneration in PD. The binding region of ASN identified in the specific amino acid region (64–100), where the region is responsible for its self-aggregation^13^. The reports state that the hydrophobic cluster formed by NAC region (85–95) and C terminus (110–130) residues. These regions are also crucial for the aggregation mechanism of ASN^14^. Currently, there are no therapeutic agents to prevent the formation of protein aggregation thus it is a main concern in this PD research field.

Xiao-Huan Li *et al*. reported a biological evaluation of DHF molecule that suppressed ASN expression and oxidative stress against MPTP induced Parkinson mice model^15^. The 7,8-Dihydroxyflavone (DHF) is a member of the flavonoid family, and highly present in vegetables and fruits. The metabolite forms of flavonoids are promoted to enhance memory and knowledge through their interfaces with neuronal signal pathways, which is essential for controlling long-term potentiation and memory in human subjects^16^. Thus reducing chances of RNS and ROS protect from neurodegenerative disorder including PD’ as oxidative stress plays a role in degeneration of neurons in PD^17–19^. In recent studies, DHF is thought to be a promising therapeutic agent for various neurodegenerative diseases^20–23^. However, only a few of the synthesized molecules have shown potent biological activity^24,25^. The 9-Fluorenylmethoxycarbonyl (Fmoc) is protecting group used in experimental peptide synthesis to keep the amino group for the further chemical reaction^26,27^. The fluorenyl ring of Fmoc forms the hydrophobic and π-π stacking interactions with reactive molecules due to its hydrophobicity and aromaticity. Moreover, the physicochemical properties of Fmoc-modified or conjugated amino acids and short peptides is significantly varied and it is used in the various applications including antibiotics, catalysis, therapeutic, drug delivery, cell cultivation, optical devices and templating^28–31^. Considering the potential pharmaceutical applications, the DHF derivatives would produce value added product and inhibit the ASN aggregation. Therefore, the present study aims to design novel potent DHF derivatives, which are linked with Fmoc-amino acids and find out the potential neuroprotective agents against human ASN using molecular docking and molecular dynamics simulations.

## Results and Discussion

### Designed DHF derivatives (Ligands)

**Figure 1:**
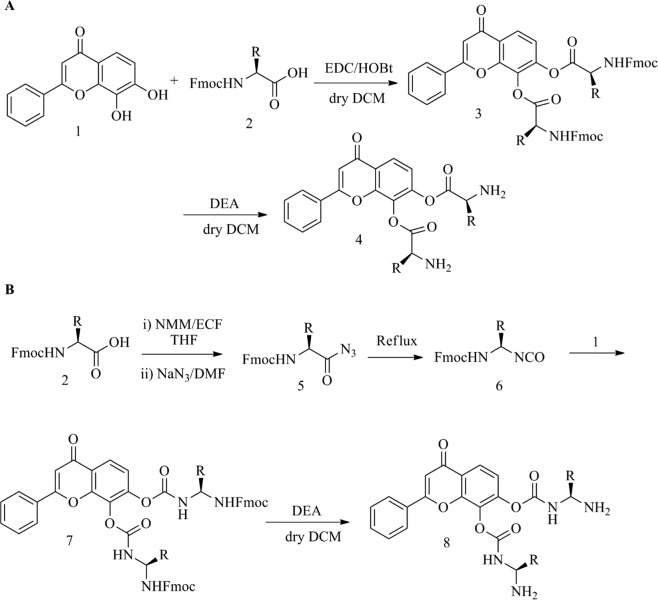
The DHF derivatives designed (**A**) Amino acid esters and (**B**) Carbamate esters.

The DHF has been linked with Fmoc-amino acid to produce amino acid ester of DHF [1 to 4 (**4a-4t**)] and carbamate esters of DHF [1, 2, 5–8 (**8a-8t**)] (Fig. 1 and Supplementary Table S1). Both the phenolic OH groups of DHF reacted with an *in-situ* generated Fmoc-amino acids and isocyanate followed by the cleavage of Fmoc-unit to afford the target conformed. The DHF hydrogen atoms of hydroxyl groups substituted with 20 different Fmoc-amino acids (alanine, arginine, asparagine, aspartic acid, cysteine, glutamine, glutamic acid, glycine, histidine, isoleucine, leucine, lysine, methionine, phenylalanine, proline, serine, threonine, tryptophan, tyrosine and valine) to produce DHF derivatives.

### Molecular docking

**Figure 2:**
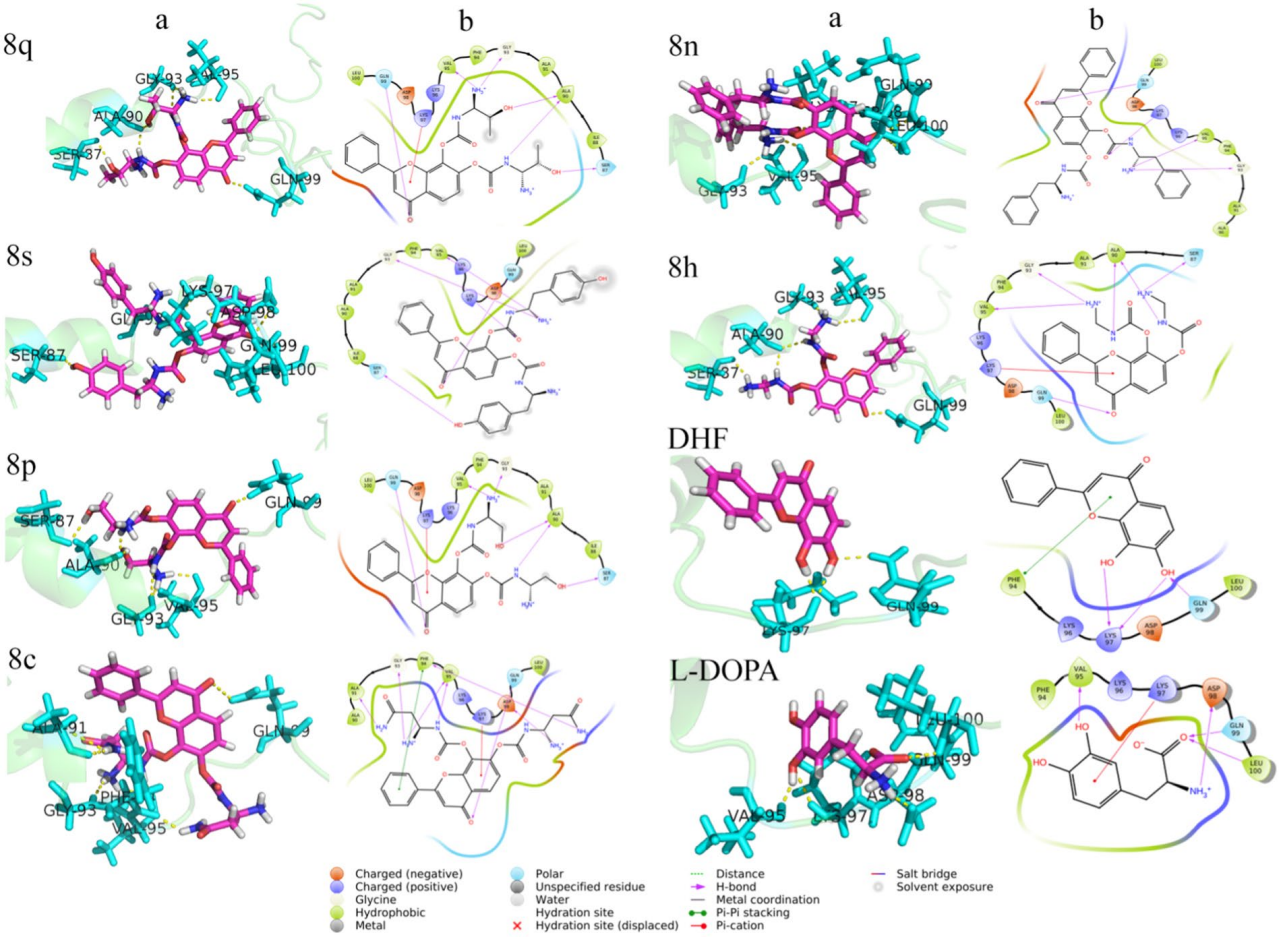
Intermolecular interactions between DHF derivatives with ASN protein, (a) 3D and (b) 2D view of 8q, 8s, 8p, 8c, 8n, 8h, DHF and L-DOPA intermolecular interaction with ASN.

Molecular docking (MD) plays a vital role in the field of computer-aided drug design. This study helps to identify the small molecules by docking towards the binding site of the protein. The ProToss analysis (build in the program) helps to predict the hydrogen bond network in the active site also could increase the number of hydrogen bonds, which hikes the scoring function^32^. Here, LeadIT suite was used for FlexX scoring function to identify the first best 200 poses based on the scoring function. To evaluate the final poses, the scoring function used HYDE function^33^. The primary purpose of the study is to find out the inhibition of a specific protein based on their score value^34^. For ASN protein, the newly designed ligands were virtually screened along with DHF and L-DOPA; and their binding energies were calculated. The designed structures and their score values are given in Supplementary Fig. S1. The molecules were chosen for further investigation based on the binding energy values. The intermolecular interactions between the ASN protein and ligands (**8q**, **8s**, **8p**, **8c**, **8n**, **8h**, DHF and L-DOPA) are shown in Fig. 2. Moreover, the conventional hydrogen bonds, van der Waals and carbon-hydrogen bonds played a significant role to maintain the stability of the complexes. The Lys97 formed Pi-cation and Pi-lone pair interactions with all molecules rather than DHF; the DHF forms two hydrogen bonds with the Lys97. The L-DOPA forms one hydrogen bond and one salt bridge with Lys97. The **8c** and L-DOPA compounds form a salt bridge bond with Asp98. Similarly, the amino acid Leu100 forms Pi-alkyl interaction with the ligands rather than **8n** and L-DOPA; these two compounds altered their interactions resulting in a strong hydrogen bond. Generally, the Val95, Gly93 and Gln99 residues form hydrogen bonding interactions with all the above said ligands, but DHF does not have hydrogen bonding interaction with Val95 and Gly93 residues. The **8c** form unfavorable donor-acceptor interaction with Val95 and Gly93; these hydrogen bonds were diminished in L-DOPA; apart from this, each molecule solidly have two hydrogen bonds with the same residue. The **8q** showed more binding capacity than other molecules. The best pose for each molecule was taken to analyze the intermolecular interactions. In the present study, **8q** showed high binding energy (−16.312 kcal/mol) and also interacts with Gln99, Ala90, Gly93, Ser87, Val95, Gly93, Lys96, Leu100 and Lys97. The **8s** (−16.188 kcal/mol) molecule interacts with Lys97, Lys96, Leu100, Gln99, Gly93, Ser87, Val95, Ala90 and Ala91. Similarly, the **8p** (−15.222 kcal/mol) interacts with Gln99, Ala90, Gly93, Ser87, Val95, Lys97, Lys96 and Leu100. The **8c** (−14.312 kcal/mol) molecule interacts with the residues Gln99, Asp98, Val95, Phe94, Ala90, Ala91, Gly93, Lys97, Phe94 and Leu100. Further, the **8n** (−14.289 kcal/mol) interacts with Lys97, Gln99, Leu100, Val95, Gly93, Lys96, Ala90 and Ala91. The **8h** (−14.281 kcal/mol) interacts with Ser87, Gln99, Ala90, Val95, Gly93, Lys97 and Leu100. **DHF** (−14.038 kcal/mol) interacts with Gln99, Lys97, Phe94 and Leu100. The **L-DOPA** (−9.156 kcal/mol) interacts with Asp98, Phe94, Gln99, Leu100, Val95, Phe94 and Lys97 of ASN. All these molecules form more than 5 hydrogen bonding interactions with the active site amino acid residues. All the molecules showed the highest negative LeadIT score, which reveals that the compounds are capable of binding with ASN to inhibit the fibrillation. Previous reports state that NAC-region (65–100) of ASN is the primary target to inhibit the aggregation process^6^. Moreover, our previous study demonstrated that CNB-001 (Docking score: −13.6158 kcal/mol) showed potent inhibitory effect against ASN followed by, DHF (−13.0499 kcal/mol), Curcumin (−12.0386 kcal/mol), Naringenin (−11.1311 kcal/mol) and emodin (−8.8539 kcal/mol). *In silico* and animal studies showed that CNB-001 diminished the expression of ASN against MPTP induced Parkinson model^35^. Interestingly, our reports revealed that DHF derivative molecules (**8q**, **8s**, **8p**, **8c**, **8n and 8h**) showed better results when compared with DHF and L-DOPA; their docking scores values are listed in Supplementary Table S3 (−16.3120, −16.1875, −15.2223, −14.3118, −14.2893, −14.2810, −14.0383 and −9.1560 kcal/mol). The interaction distances of each molecule with their respective active site amino acid residues are shown in Supplementary Table S2.

### ADMET investigation

The ADMET profile was found to play a vital role to understand the pharmacokinetic properties of the ligand molecules for therapeutic intervention. In this analysis, we revealed that the drug likenesses of selected compounds studied by calculating the ADMET properties using “TOPKAT” module of DS 3.5. The compounds (**8q**, **8s**, **8p**, **8c**, **8n**, **and 8h**) were chosen based on Veber’s rule^36^, which satisfies the molecular weight (~500 g/mol) of all drugs. The compounds were investigated using Lipinski’s rule of five^37^; in which, the H-bond donors and acceptors were counted. The counting of H-bond is based on the electronegative atoms present in the drug molecules. All the compounds have ~6 donors and ~8 acceptors, and the ratio of compound concentration in a mixture of two immiscible phases of partition-coefficient (logP). The logP values of the ligand molecules were an acceptable threshold (<5) level (Supplementary Table S3). The AQ SOL LEV prediction tool was used to investigate the biological transportation functions of the drug molecules through a water-based solvent, the values are lies between 2 to 3 except L-DOPA (5).

Further, BBB LEV analysis reveals the information about drugs passing through the blood-brain barrier and their values were calculated based on logP, AQ SOL LEV and lipophilic characters. The BBB LEV value of the molecules are high (4) on compared with DHF (2). The critical parameters, such as Plasma Protein Binding levels (PPB LEV) and Hepatotoxicity (HEPATOX) with CYP2D6 scores were calculated from ADMET properties. The values of CYP2D6 scores were found to be less than 0, which shows that the molecules are a non-inhibitory function to the Cytochrome P450 2D6 enzyme; whereas CYP2D6 ≥ 1 shows that they are inhibitory. Calculated PPB LEV provides the detail of drug concentration and an insight into the plasma protein. PPB LEV with high values paves the way for the adsorption of the drug in the renal passage and prevents PD. All the compounds exhibited an excellent binding capacity and diffuse through the membrane, which also confirms the high levels of PPB. The hepatotoxicity levels were predicted, to study the molecular toxicity of the organ. When the HEPATOX value is 1, then the molecules could be highly toxic; on the other hand, if the value is 0, then they are non-toxic. The HEPATOX value of the molecule is < 0 shows that they are non-toxic. In the present study, NTP carcinogenicity, mutagenicity and developmental toxicity were performed to predict the toxicity profiles of the molecules. The skin irritation studies include a topical application for skin or mucous membranes. TOPKAT features is a patented algorithm (US Patent 6,036,349, issued March 14, 2000), which decides whether the compound lies within the optimum prediction space (OPS) for toxicity analysis and skin irritation studies. If the range of OPS is lies between 0 to 0.29, the compound belongs to the non-toxic group. Consequently, the range is between 0.3 to 0.69 they are indeterminate and the score lies between 0.7 to 1, the molecule is considered to be highly toxic. The OPS score (1.0) of **8c** is high; which indicates that it is highly toxic for the skin. On the other hand, the molecules **8q**, **8s**, **8p**, **8n**, **8h**, DHF and L-DOPA exhibits low OPS score (0.0, 0.0, 0.247, 0.0, 0.0, 0.0 and 0.303). Further, the AMES mutagenicity prediction analysis has been carried out, it shows that all the molecules are non-mutagenic except **8** **h**.

### Molecular dynamics

The molecular dynamics (MD) simulation was performed to find out the stability, confirmation and intermolecular interaction of the ligand molecules with ASN protein. The time-dependent modification of the complexes was calculated over 50 ns using Desmond package. The MD simulation was performed under the thermodynamical conditions (applied volume, density, pressure and temperature). The complete system was annealed and equilibrates using ensembles. Moreover, the final production step performed to investigates the structural modification of the complex. Further, the trajectories of each complex subjected to specific parameters such as root mean square deviation (RMSD), root mean square fluctuation (RMSF), protein secondary structure element (SSE), conformational modification of ligands and intermolecular interactions to analyze the level of structural changes.

### RMSD and RMSF

**Figure 3:**
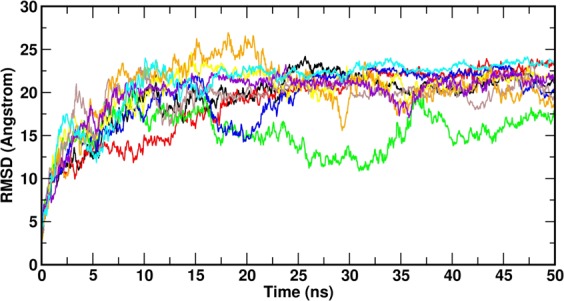
The RMSD plot for all complexes during the MD simulation. (**8q**–red, **8s**–green, **8p**–blue, **8c**–yellow, **8n**–brown, **8h**–orange, DHF–meganta, L-DOPA–cyan and ASN alone–black).

The backbone deviation (N, Cα, C) of protein was calculated from the RMSD value during the MD simulation. The complex structures were highly fluctuated (1 to 27 Å) up to equilibration. After equilibration (5 ns), the system gets stabilized, and this trend continued up to 50 ns. After 5 ns, the RMSD of the complexes varied ~1 to 4 Å. Notably, the RMSD of DHF and its derivatives are low on compared with L-DOPA. In which, the RMSD of **8q**, **8p**, **8c**, **8n**, **8h** and DHF is not much varied except **8s**. It indicates that the molecules were highly stable during the MD simulation (Fig. 3). The RMSD analyzes confirm that the DHF derived molecules **8q**, **8p**, **8c**, **8n**, **8h** and DHF showed structural stability during the MD simulation.

**Figure 4:**
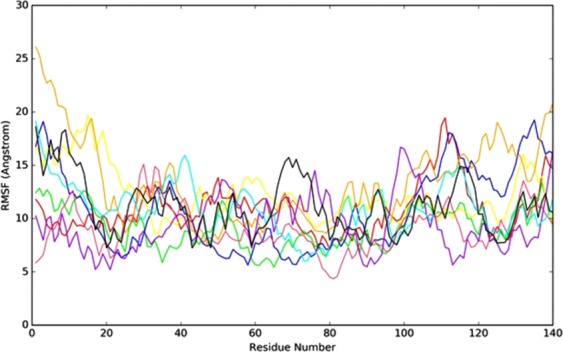
The RMSF plot for all complexes during the MD simulation. (**8q**–red, **8s**–green, **8p**–blue, **8c**–yellow, **8n**–brown, **8h**–orange, DHF–meganta, L-DOPA–cyan and ASN alone–black).

Further, the flexibility of the complexes was analyzed, while the ligands present in the active site of the protein. The RMSF was used to investigate the fluctuation of the complexes in the function of time. The N-terminal (~25 Å) has high fluctuation compared with C terminal (~22 Å). The DHF derivative molecules were highly stable in the catalytic region except for **8n**, due to the weak intermolecular interaction with the protein (Fig. 4). The complexes exhibit low fluctuation in NAC region (64–100); it is due to the intermolecular interactions of the ligand molecules with ASN. Moreover, the intermolecular interactions and secondary structure elements (alpha helices and beta strands) make the protein molecule is slightly rigid.

### Protein secondary structure elements (SSE)

The SSE (alpha-helices and beta-strands) monitored throughout the simulation process. Supplementary Figs. S2 and S3 describe the SSE distribution and SSE composition analysis of the respective residue index of the ASN. Except **8s**, **8h**, and L-DOPA complexes, other complexes have beta-strands; those complexes were highly helical nature. SSE analyzes of ASN confirms that, the secondary structure is not presented in the C-terminal region (100–140 residues). The torsion angle potential plot exhibits the relation between the torsion angle present in the ligands and their corresponding potential energy. The information of torsion angle is necessary to predict the rotatable bonds of ligand molecule (Supplementary Fig. S4). Here, **8s and 8c** each contain 15 rotatable bonds; whereas, **8q**, **8p**, **8n**, **8h**, DHF and L-DOPA contain 13, 13, 13, 9, 3 and 5 rotatable bonds respectively. The histogram and torsion potential relationships give an insight into the conformational strain of the ligands, which is used to understand the protein-bound ligand conformation. The ligand modification showed in Supplementary Fig. S5 includes RMSD, radius of gyration, intra-molecular hydrogen bonds, molecular surface area (MolSA), solvent accessible surface area (SASA) and polar surface area (PSA).

### Intermolecular interaction

**Figure 5:**
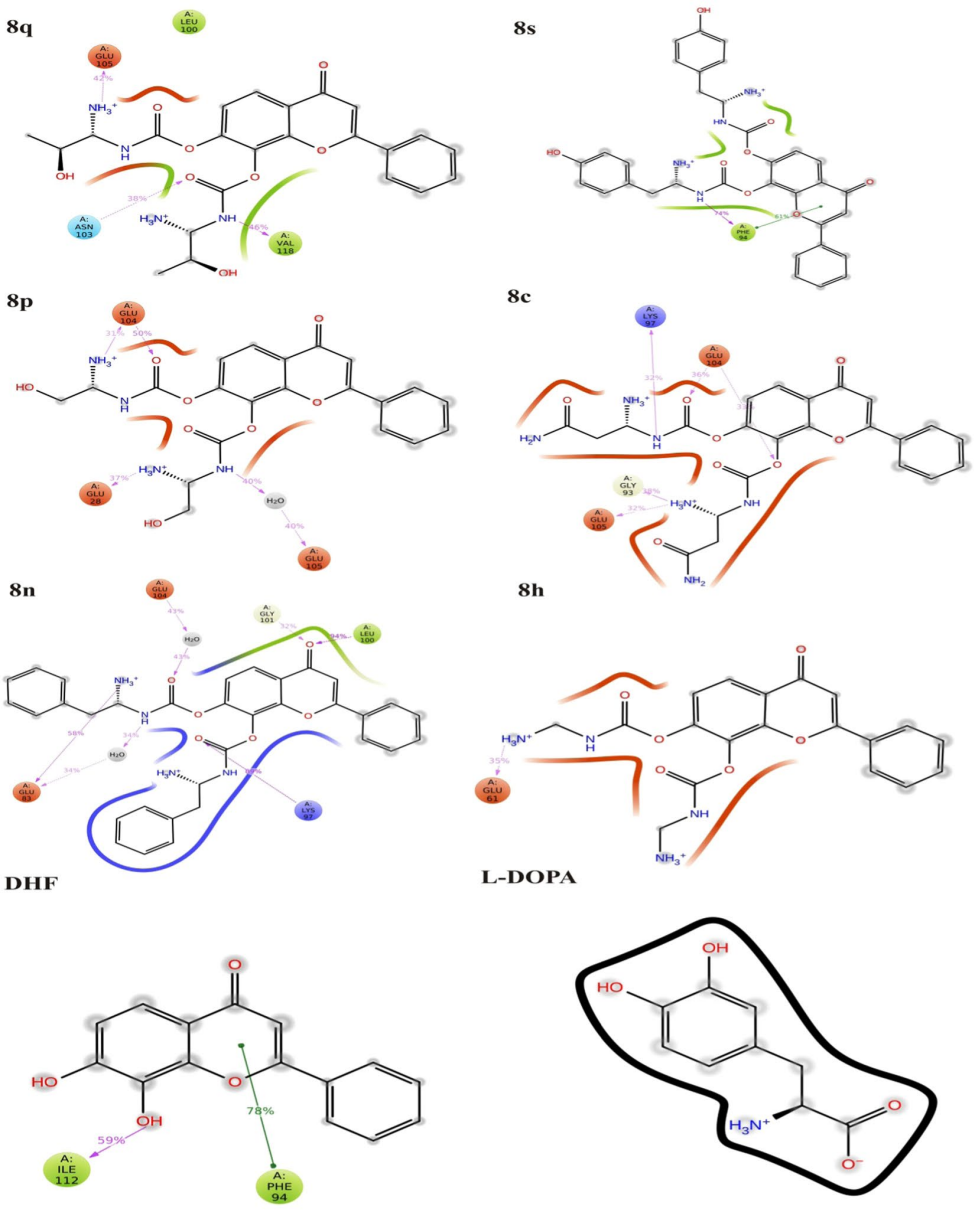
2D Intermolecular interactions of **8q**, **8s**, **8p**, **8c**, **8n**, **8h**, DHF and L-DOPA ligands with ASN protein.

**Figure 6:**
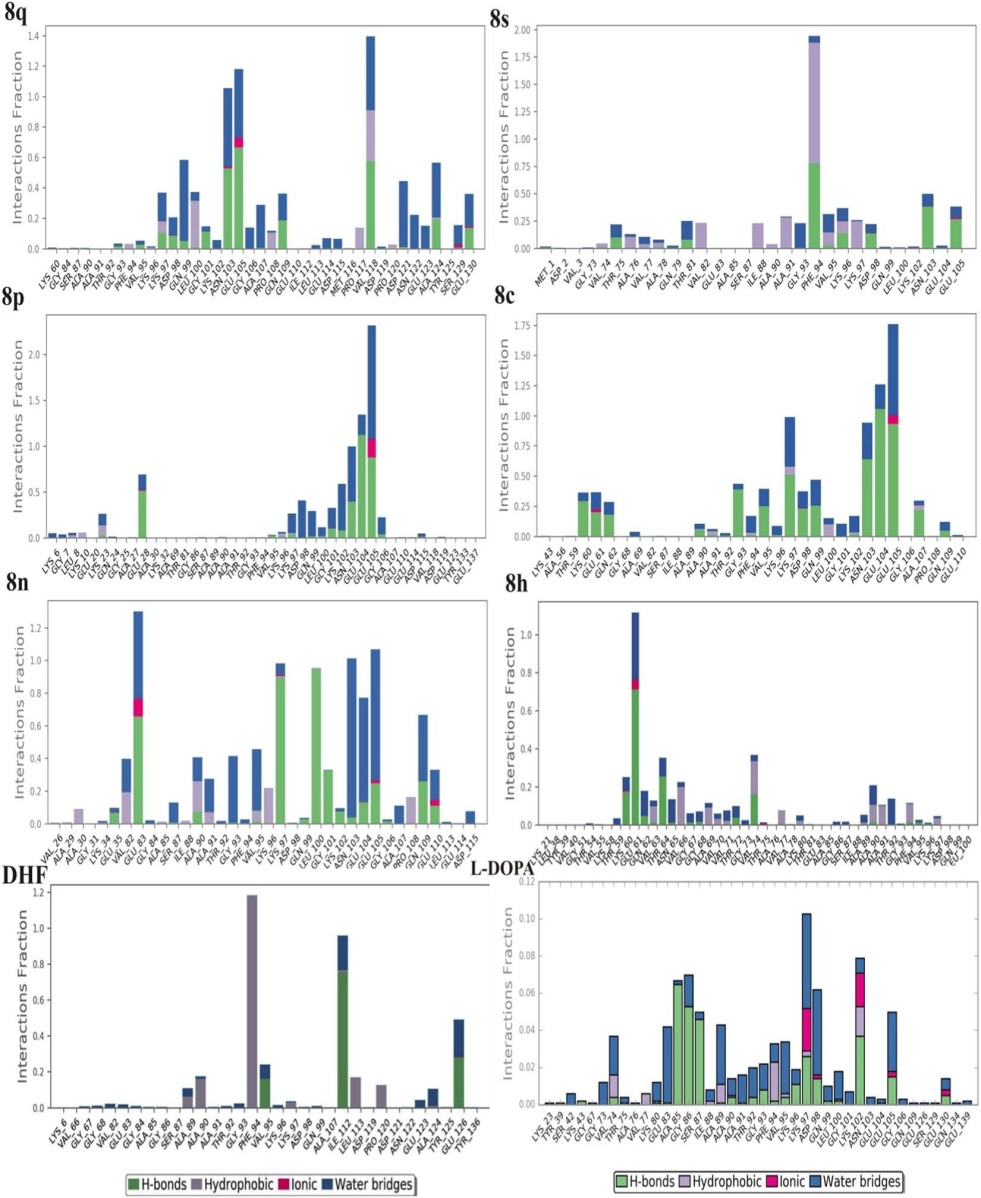
Protein-ligand interactions between ASN with respective compounds **8q**, **8s**, **8p**, **8c**, **8n**, **8h**, DHF and L-DOPA.

The atomic level information is essential to predict the binding mode of **8q**, **8s**, **8p**, **8c**, **8n**, **8h**, DHF and L-DOPA in the binding site of ASN protein. For binding mode analysis, the intermolecular interactions such as hydrogen bond, hydrophobic contact, ionic interaction and salt bridge were analyzed over 50 ns MD simulation studies. The report states that the hydrophobic cluster formed by NAC region (85–95) and C terminus (110–130) residues. These regions are also crucial for the aggregation mechanism of ASN^14^. The present study also confirms that the DHF derivatives form strong intermolecular interactions with both NAC (65–100) and C-terminal (101–140) residues. The **8q** forms strong ionic interactions with binding site residues of Glu105. Along with this, the **8q** forms hydrophobic interactions with Leu100 and Val118 by making Pi- Pi stacking wall, also one polar interaction with Asn103. The **8s** form one hydrophobic interaction with Phe94. The **8p** forms two ionic contacts (Glu28, Glu104) and one water mediated bridge bond (Glu105). Similarly, the **8c** forms ionic contact (Glu104, Glu105), polar (Gly93) and charged interaction (Lys97) with the active site residues. Likewise, the **8n** forms hydrophobic (Leu100) and three charged interactions (Glu83, Lys97 and Glu104). Further **8h** and DHF compounds display very less accountable interactions. Here, **8h** has only one charged interaction (Glu61) and DHF forms two hydrophobic interactions (Phe94 and Ile112). Whereas, L-DOPA loss the intermolecular interactions with active site residues and slightly moved away from the active site on compared with all other complexes (Fig. 5 and Supplementary Fig. S6). The reports state that the ligand molecules induce the α-helix formation of ASN^38,39^. Similarly, in the present study, the **8q** and **8s** form the possible interactions with NAC and C-terminal of ASN. In which, the **8q** molecule induces the folded state of ASN (Supplementary Fig S6), it leads to α-helix formation. This mechanism inhibits the aggregation of ASN. The protein-ligand intermolecular interactions confirm the binding strength and stability of DHF derivatives with the active site amino acid throughout the MD simulations (Fig. 6 and Supplementary Fig. S7).

## Materials and Methods

### Computational feature

The *in silico* analyses were performed using HP workstation Z220 with Next-generation 22 nm processor. The DHF derivative molecules were drawn using ChemDraw Ultra 12 (ChemDraw)^40^. Absorption, Distribution, Metabolism, Excretion and Toxicity (ADMET) properties of DHF derivative molecules were analyzed using Accelery Discovery Studio (DS) 3.5^41,42^. Further, the docking studies were carried out using Biosolve IT (Lead IT) software package^43^ and Desmond v3.6 Package was used to run the MD simulation. The intermolecular interactions were analyzed using Pymol and Chimera^44,45^.

### Designing of DHF derivatives

**Figure 7:**
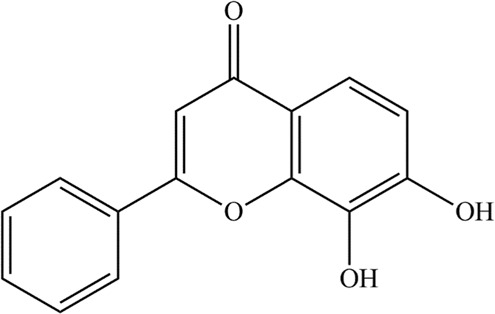
Chemical structure of 7,8-Dihydroxyflavone.

The chemical structure of DHF shown in Fig. 7. DHF derivatives designed and their structures drawn using ChemDraw software. The DHF derivatives depicted in two different schemes, each scheme of DHF reacts with 20 different Fmoc amino acids to produce 40 DHF derivatives and it is used in *in silico* analysis.

### Docking study

Molecular docking studies used to find out the binding affinity of the ligand molecule with protein. The experimental structure of ASN protein retrieved from PDB (PDB ID: 1XQ8) database^13^. In which, the water molecules were removed, followed by hydrogen atoms, charges, standard bond orders and the missing residues were added using protein preparation wizard in DS 3.5 software^46^. Minimization was carried out up to 500 steps using smart minimizer methods and the standard dynamic cascade was performed using DS 3.5 simulation protocol^47^. The ligands and protein were prepared using Ligprep and protein preparation wizard available in the DS 3.5. The refined molecules docked using LeadIT software package, which is based on the FlexX docking approach. The FlexX algorithm was used to generate up to 200 poses for each ligand; the best conformer will be scored in high. The interaction modes between the ligands (DHF derivatives) and protein were studied using Biosolve IT FlexX^48^.

### ADMET properties

The molecules were subjected to ADMET analysis using DS 3.5 protocol to predict the pharmacokinetics and toxicity properties^49,50^. The ADMET studies provide insight into the pharmacokinetics properties such as Plasma Protein Binding level (PPB LEV), Hepatotoxicity (HEPATOX), CYP 2D6, Blood Brain Barrier Level (BBB LEV) and Aqueous Solubility level (AQ SOL LEV). The toxicity profile of the compounds was predicted using TOPKAT 6.1, which uses a range of robust, cross-validated and Quantitative Structure-Toxicity Relationship (QSTR) models for identifying specific toxicological activity^51^. Toxicity profiles were tested, including NTP Carcinogenicity Call (Male Mouse) (v3. 2), NTP Carcinogenicity Call (Female Mouse) (v3. 2), Developmental Toxicity Potential (DTP) (v3. 1), Skin Irritation (v6. 1) and Ames Mutagenicity (v3. 1).

### Molecular dynamics

The MD simulations were performed using Desmond v3.6 Package to elucidate the fact behind the effectiveness of these compounds against ASN inhibition^52,53^. The lead compounds such as DHF, L-DOPA and the DHF derivatives with ASN protein were prepared using the OPLS2005 force field^54^. Further, the pre-defined TIP3P water model was used to build the system, which could act as water molecules and these are constructed in the orthorhombic periodic boundary conditions at the distances of 10 Å units^55^. Moreover, the charge of the complexes electrically neutralized with balancing Na^+^/Cl^−^ ions and also the system minimized their energies by heating and equilibrium processes before the MD simulations. The complexes were subjected to the minimization protocol based on the steepest descent method, then heated at 0–300 K with the annealing steps of 2000 and the time steps of 0.001 ps. Further, the system normalized in an equilibrium state at 1000 steps with the time step of 0.001 ps. The final production step of the system continued up to 50 ns, at the time steps of 0.001 ps; 300 K temperature and 1 Atm pressure, applied using Nose-Hoover method^56^ with NPT ensemble^57,58^. The best conformations were selected based on the interactions and dynamical properties of the complexes^59^.

## Conclusion

In this study, 40 different DHF derivatives were designed through the amino acid esters and carbamate esters. Among the 40 DHF derived complexes, the molecule **8q** exhibited the highest molecular docking score (−16.3120 kcal/mol), indicates that it has high binding towards ASN. The ADMET properties reveal that **8q**, **8s**, **8p**, **8c**, **8n**, **8h**, DHF and L-DOPA compounds are non-toxic. The stability of the ligand-protein complexes were evaluated from the molecular dynamics simulation showed that the ligands **8q**, **8s**, **8p**, **8c**, **8n**, **8h** and DHF with ASN complexes have subtle structural modification throughout the MD simulations. From the binding mode analysis, it is confirmed that the DHF and its derivatives are forming stable interactions with the ASN protein except L-DOPA. The DHF derivatives **8q**, **8s**, **8p**, **8c**, **8n** and **8h** potentially inhibit the ASN when compared with DHF and L-DOPA; among these, presumably, **8q** molecule may be the potential candidate to inhibit the ASN aggregation. On the whole, these compounds of novel scaffolds provide valuable leads for further optimization in both *in vitro* and *in vivo* as potent inhibitors against ASN to treat PD. In conclusion, these results suggest that the carbamate ester of DHF showed better efficiency than the L-DOPA.

## Supplementary information

Supplementary information.
