# Author Correction: Design and Molecular dynamic Investigations of 7,8-Dihydroxyflavone Derivatives as Potential Neuroprotective Agents Against Alpha-synuclein

**DOI:** 10.1038/s41598-020-61862-x

**Published:** 2020-03-16

**Authors:** Thangavel Mohankumar, Vivek Chandramohan, Haralur Shankaraiah Lalithamba, Richard L. Jayaraj, Poomani Kumaradhas, Magudeeswaran Sivanandam, Govindasamy Hunday, Rajendran Vijayakumar, Rangasamy Balakrishnan, Dharmar Manimaran, Namasivayam Elangovan

**Affiliations:** 1 Department of Biotechnology, School of Biosciences, Periyar University, Salem, 636011 Tamilnadu India; 2 Department of Biotechnology, Siddaganga Institute of Technology, Tumakuru, 572103 Karnataka India; 3 Department of Chemistry, Siddaganga Institute of Technology, Tumakuru, 572103 Karnataka India; 4 Department of Pharmacology and Therapeutics, College of Medicine and Health Sciences, Al-Ain, Abudhabi 17666 United Arab Emirates; 5 Department of Physics, School of Physical Sciences, Periyar University, Salem, 636011 Tamilnadu India; 6 Department of Biology, College of Science in Zulfi, Majmaah University, Majmaah, 11952 Saudi Arabia

Correction to: *Scientific Reports* 10.1038/s41598-020-57417-9, published online 17 January 2020

The original version of this Article contained errors in the spelling of the authors Thangavel Mohankumar, Haralur Shankaraiah Lalithamba, Poomani Kumaradhas, Magudeeswaran Sivanandam, Govindasamy Hunday, Rangasamy Balakrishnan, Dharmar Manimaran and Namasivayam Elangovan, which were incorrectly given as Mohankumar Thangavel, Lalithamba Haralur Shankaraiah, Kumaradhas Poomani, Sivanandam Magudeeswaran, Hunday Govindasamy, Balakrishnan Rangasamy, Manimaran Dharmar and Elangovan Namasivayam.

As a result, the Author Contribution section now reads:

“T.M., V.C., H.S.L. and N.E. designed the research, T.M., V.C., P.K., M.S., G.H. and N.E. performed the in silico study and analyzed the results, T.M., V.C., R.L.J., R.V., R.B., D.M. and N.E. prepared the manuscript. All the authors reviewed the manuscript”

This has now been corrected in the PDF and HTML versions of the Article, and in the accompanying Supplementary Information file.

