# Supplementary material for: Design and Molecular dynamic Investigations of 7,8-Dihydroxyflavone Derivatives as Potential Neuroprotective Agents Against Alpha-synuclein
Source: Sci Rep. 2020 Jan 17;10:599. doi: 10.1038/s41598-020-57417-9 (PMC6969171; doi:10.1038/s41598-020-57417-9)
Supplement: Supplementary file 1 — Supplementary information. [file 41598_2020_57417_MOESM1_ESM.pdf]

SUPPLEMENTARY INFORMATION

Design and Molecular dynamic Investigations of 7,8-Dihydroxyflavone Derivatives as Potential Neuroprotective Agents Against Alpha-synuclein

Mohankumar Thangavel[a], Vivek Chandramohan[b], Lalithamba Haralur Shankaraiah[c], Richard L Jayaraj[d], Kumaradhas Poomani[e], Sivanandam Magudeeswaran[e], Hunday Govindasamy[e], Rajendran Vijayakumar[f], Balakrishnan Rangasamy[a], Manimaran Dharmar[a], Elangovan Namasivayam[a,*]

[a]Department of Biotechnology, School of Biosciences, Periyar University, Salem, Tamilnadu, India.

[b]Department of Biotechnology, Siddaganga Institute of Technology, Tumkur, Karnataka, India.

[c]Department of Chemistry, Siddaganga Institute of Technology, Tumkur, Karnataka, India.

[d]Department of Pharmacology and Therapeutics, College of Medicine and Health Sciences, Al-Ain, Abudhabi, 17666, United Arab Emirates.

[e]Department of Physics, School of Physical Sciences, Periyar University, Salem, Tamilnadu India.

[f]Department of Biology, College of Science in Zulfi, Majmaah University, Majmaah, 11952, Saudi Arabia.

Corresponding author E-mail: elangovannn@gmail.com

This PDF file includes:

Supplementary text

Supplementary Figure 1-7

Supplementary Table 1-3

List of Supplementary figures

8q (-16.3120)

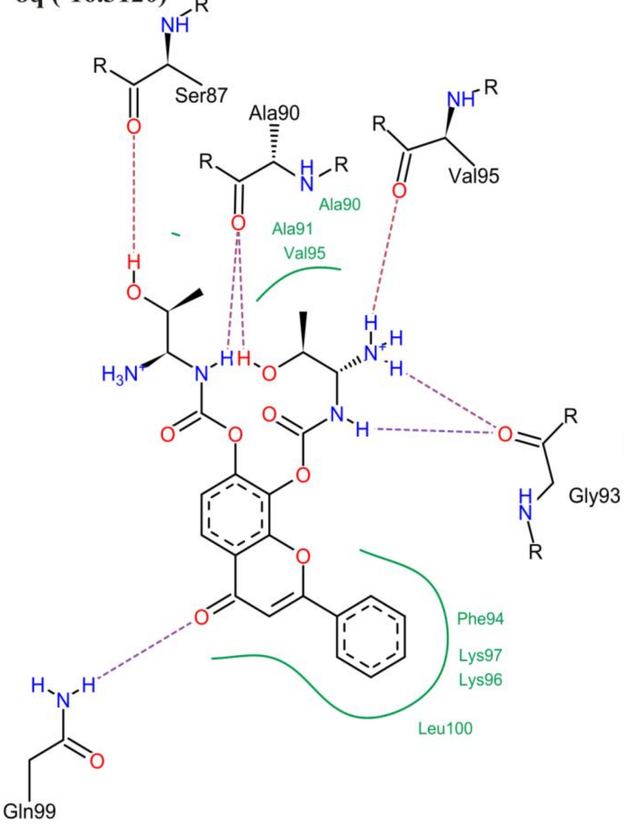

8s (-16.1875)

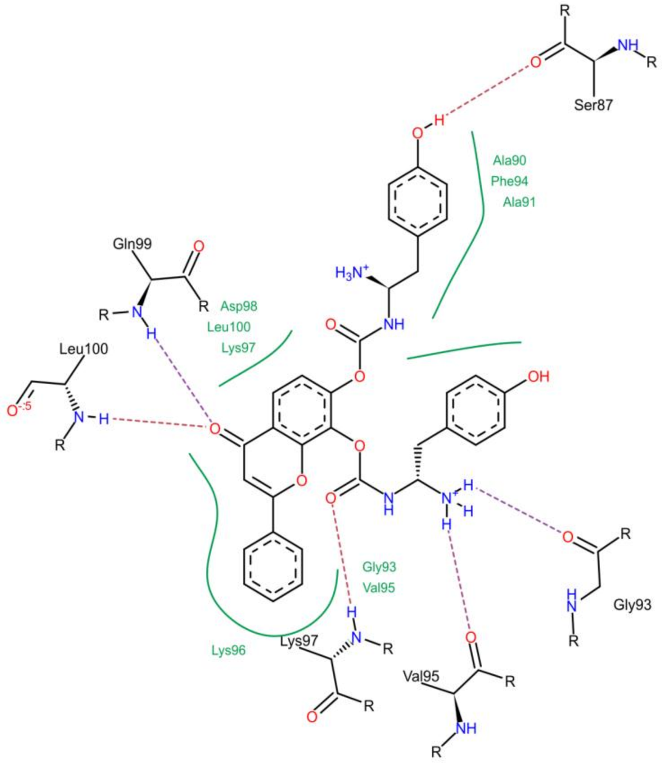

8p (-15.2223)

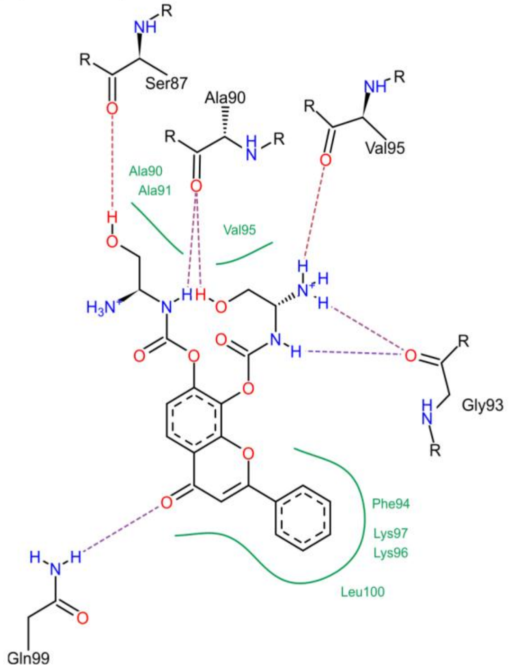

8c (-14.3118)

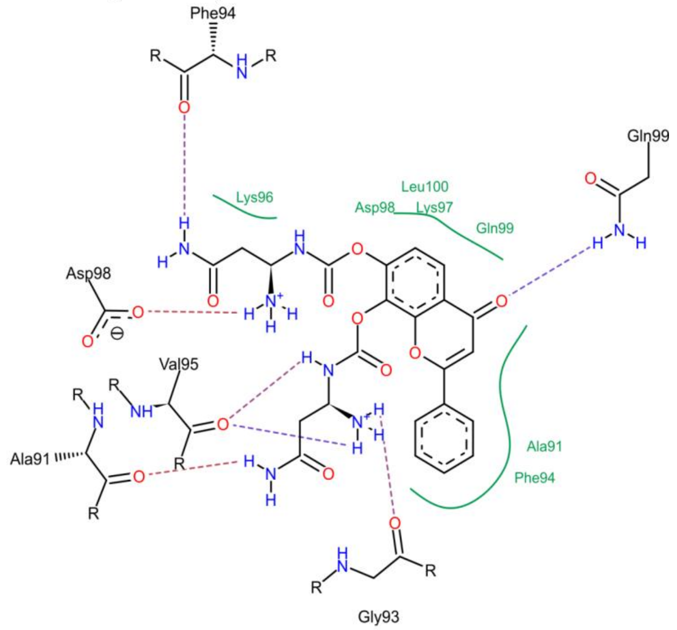

8n (-14.2893)

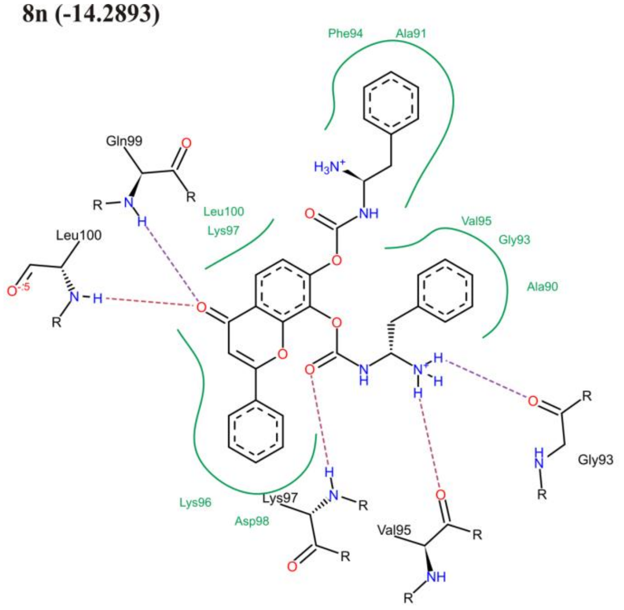

8h (-14.2810)

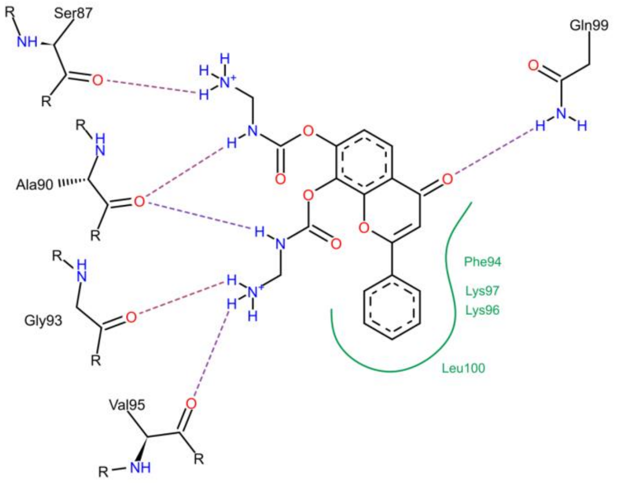

8i (-13.9648)

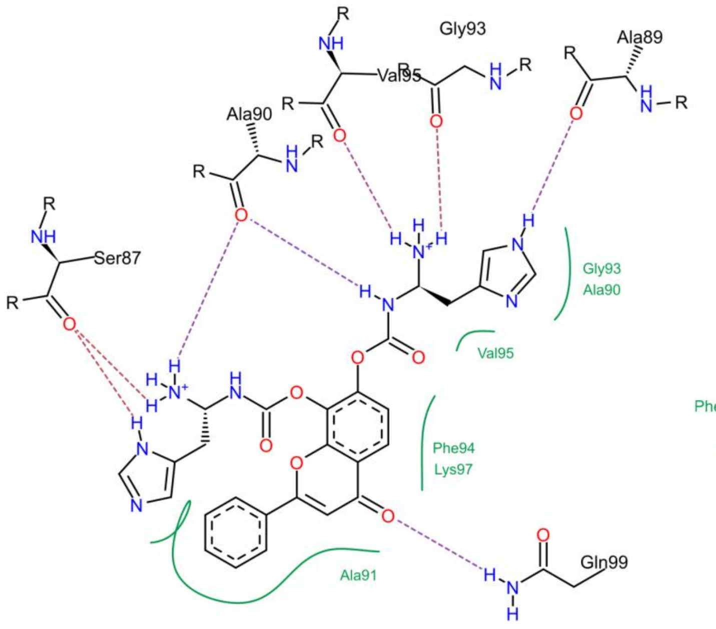

8o (-13.5546)

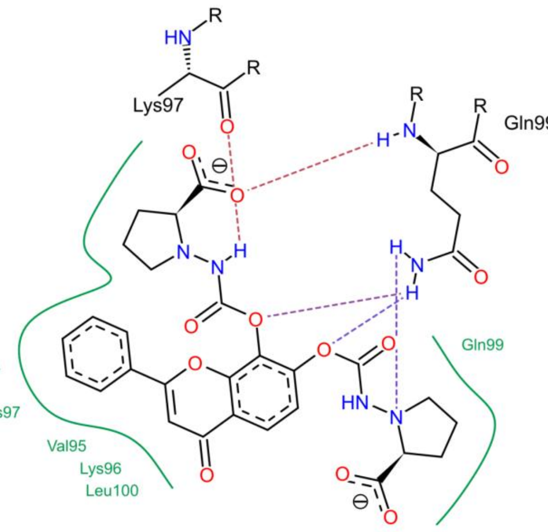

8r (-13.0428)

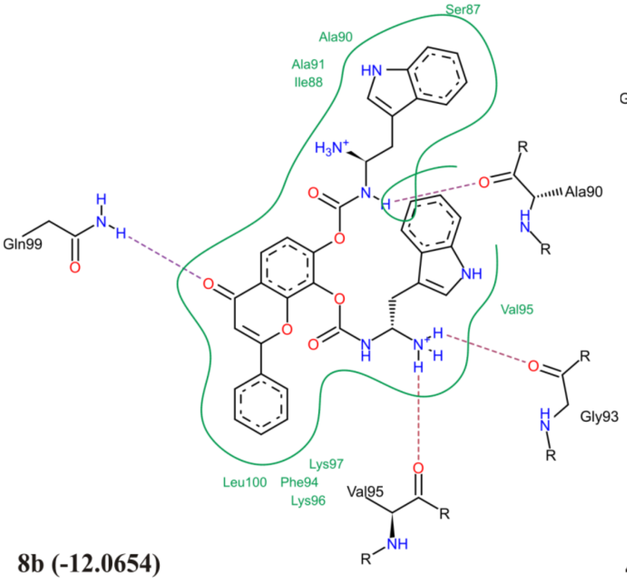

8d (-12.4647)

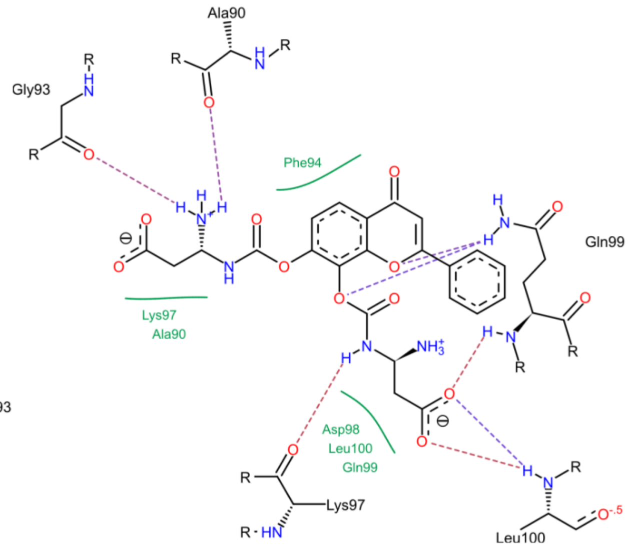

8b (-12.0654)

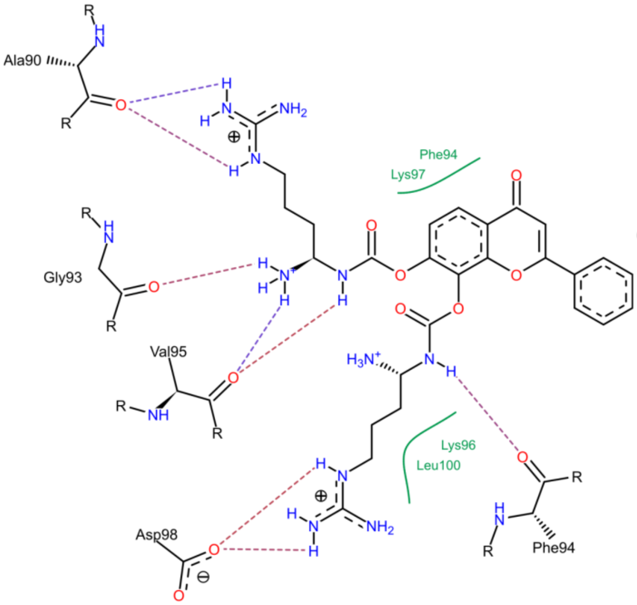

4b (-11.8605)

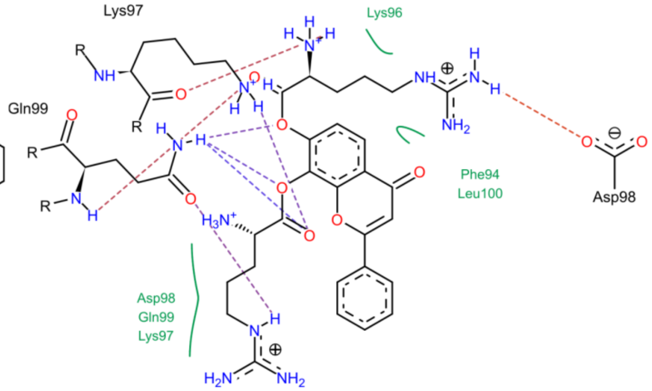

8a (-11.6654)

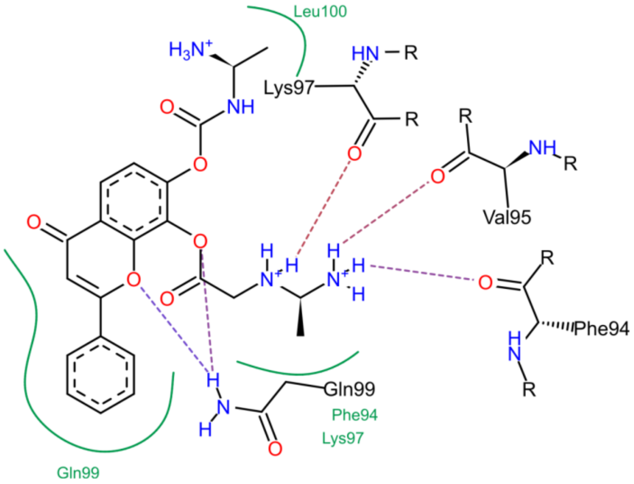

8f (-11.4819)

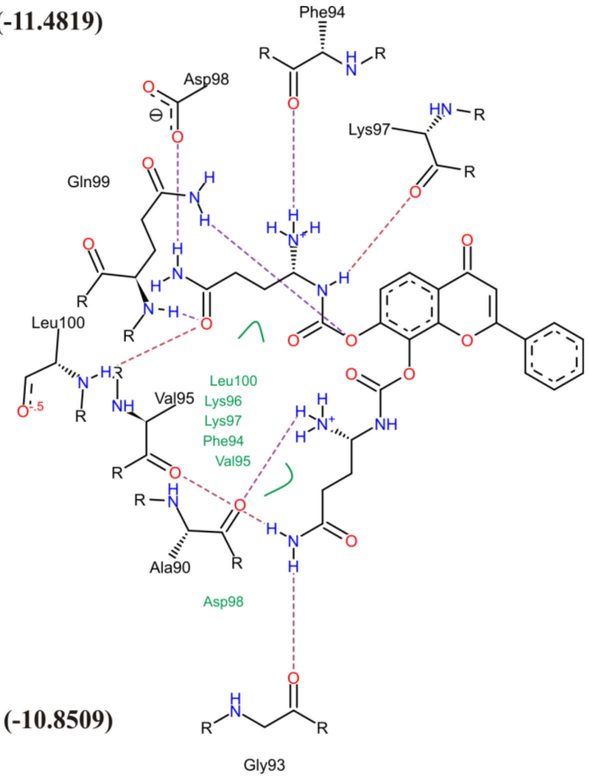

8g (-11.0592)

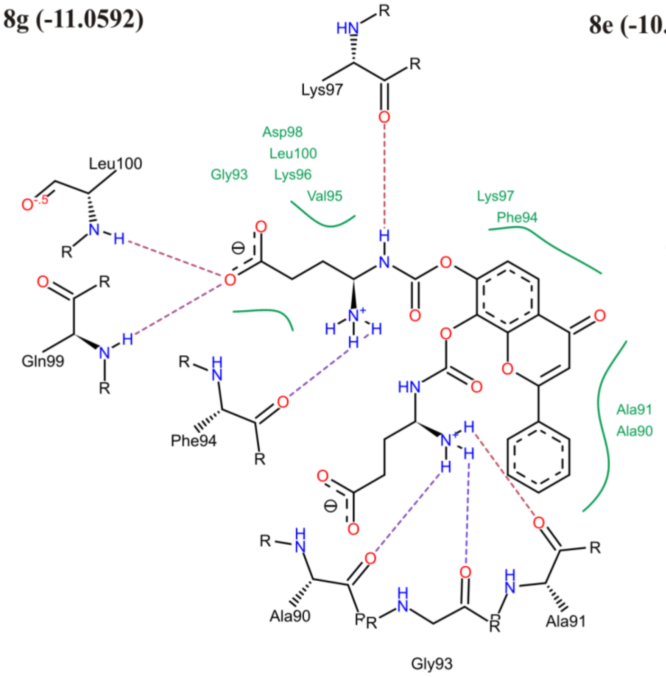

8e (-10.8509)

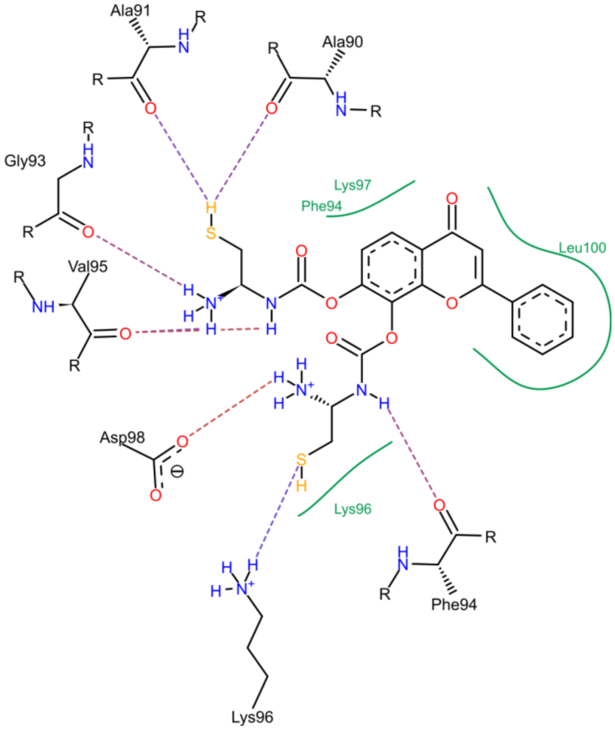

4o (-10.5688)

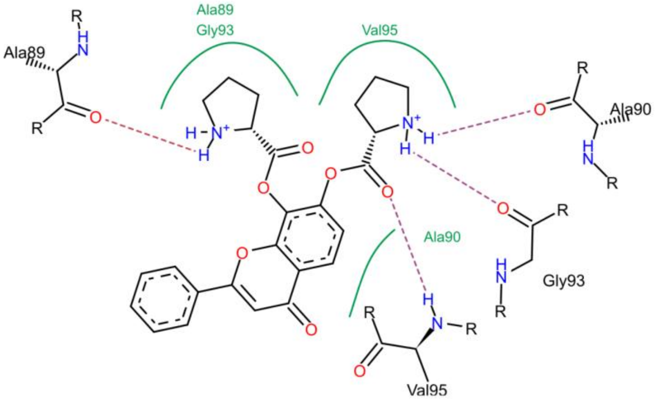

8j (-10.3430)

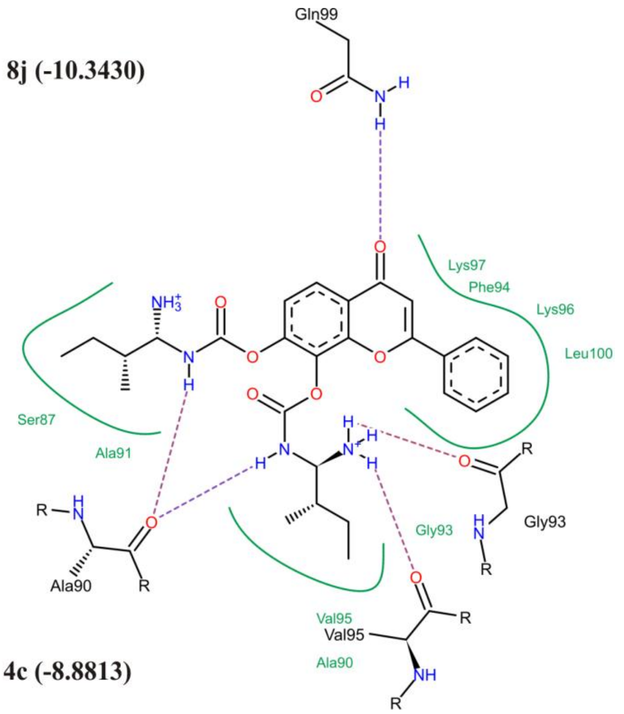

4p (-10.0120)

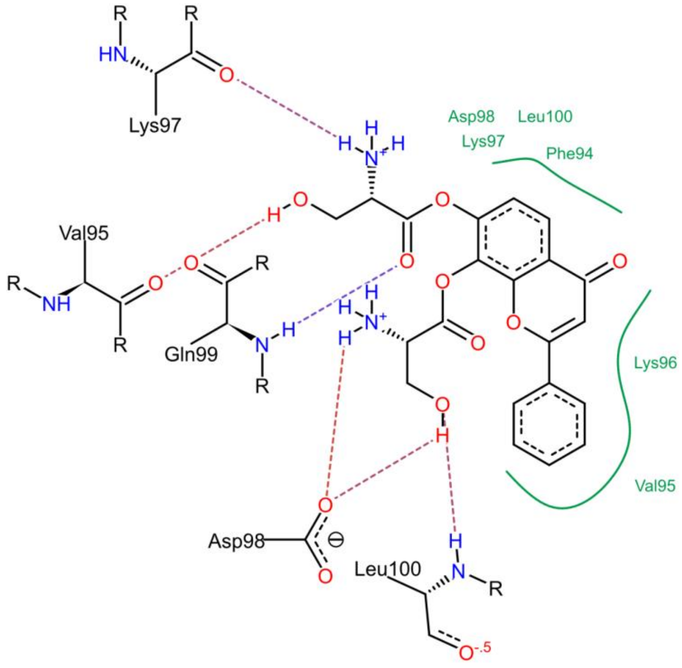

4c (-8.8813)

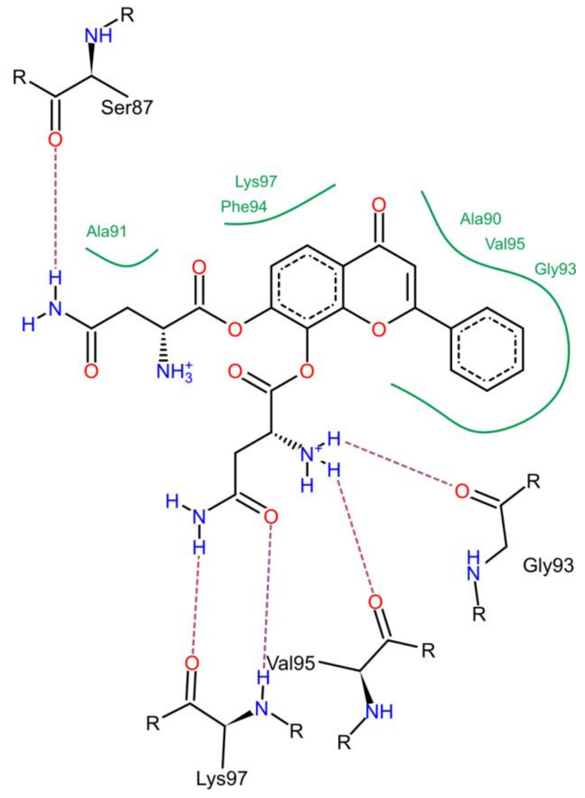

8t (-8.7657)

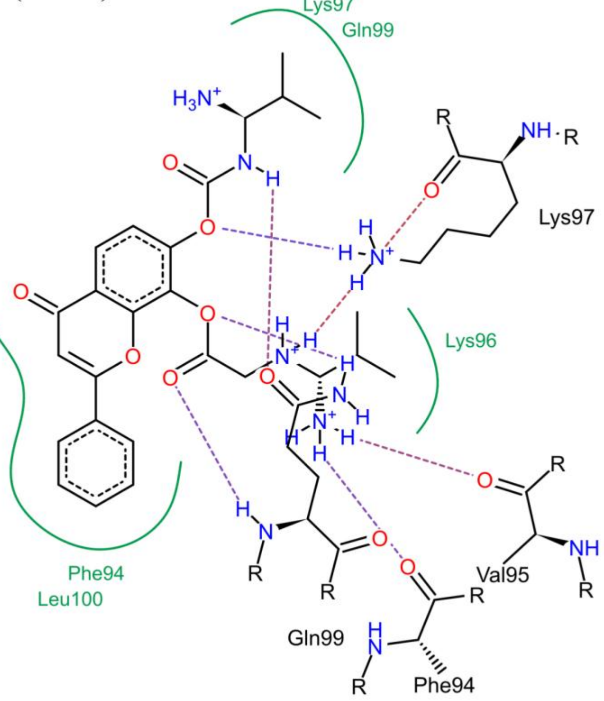

8k (-7.6249)

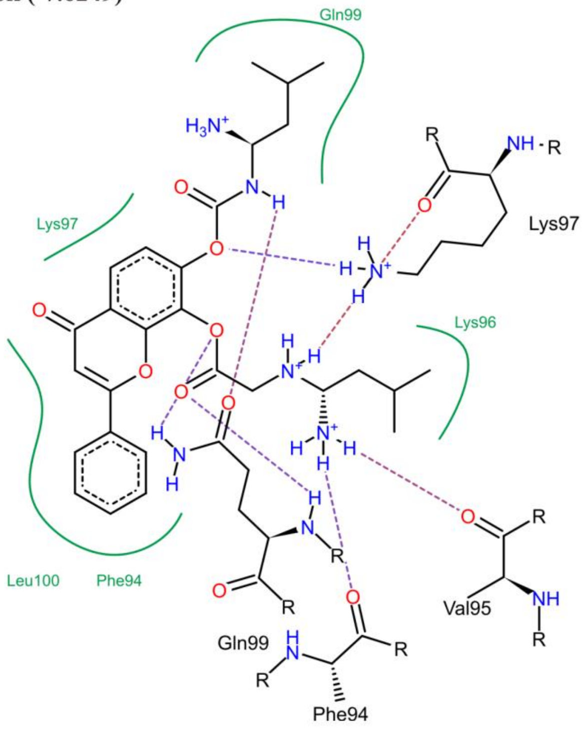

4r (-7.5145)

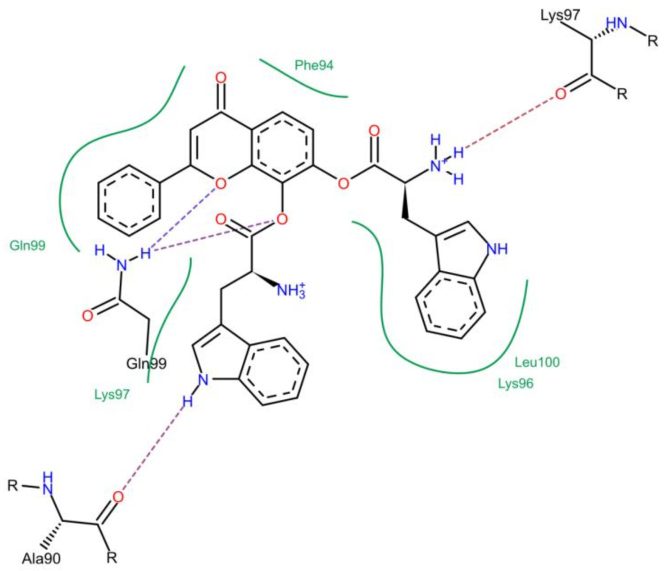

4i (-7.0715)

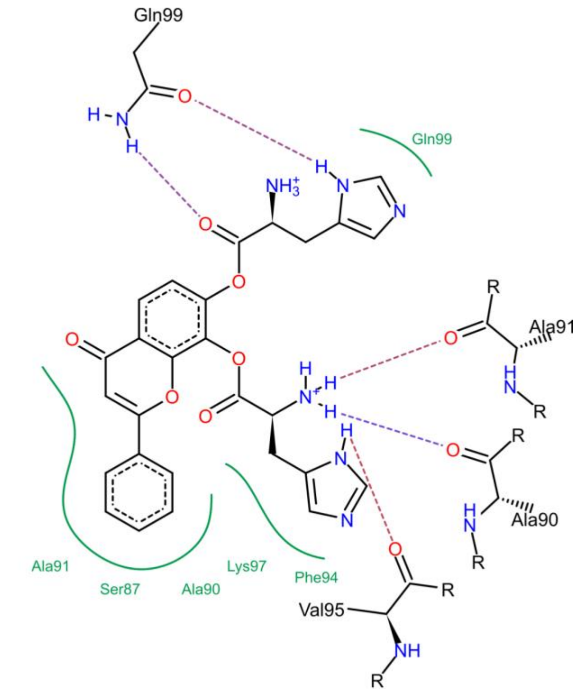

8l (-6.4282)

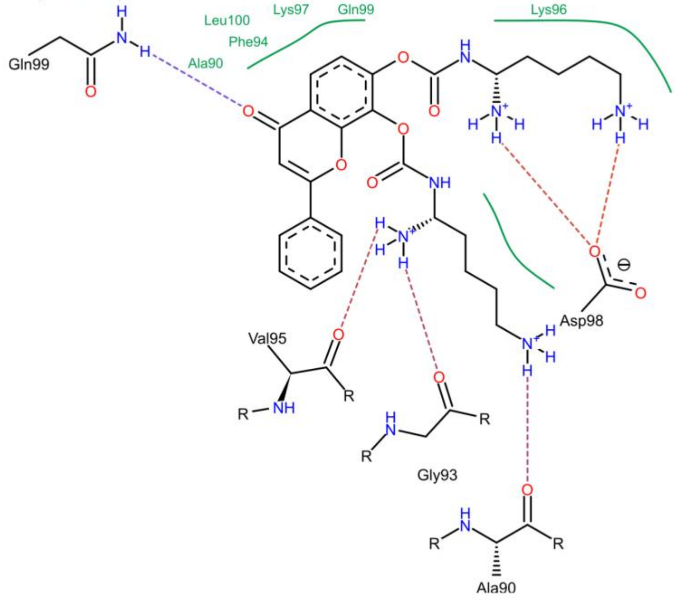

4a (-5.2385)

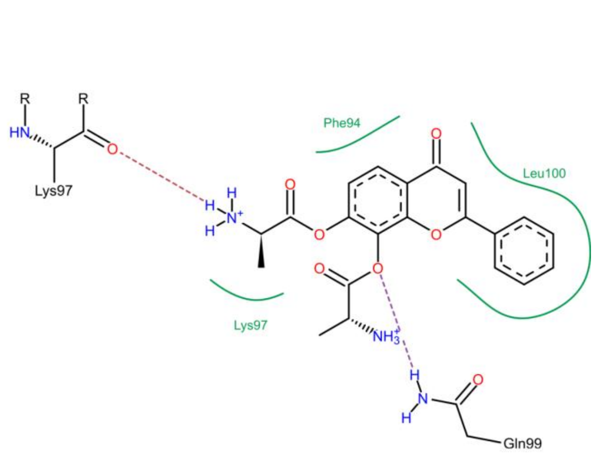

8m (-4.8384)

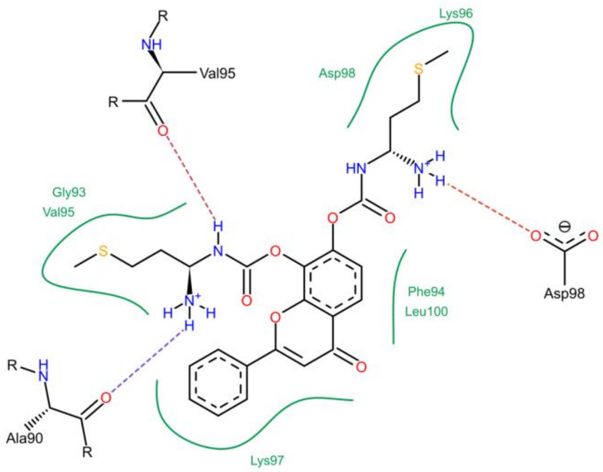

4f (-4.0678)

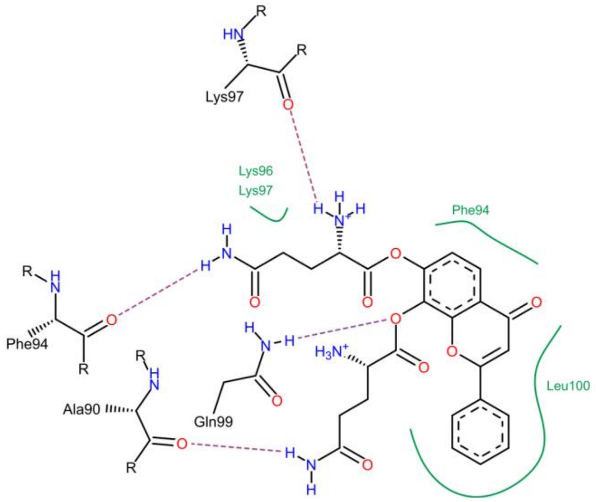

4n (-3.6590)

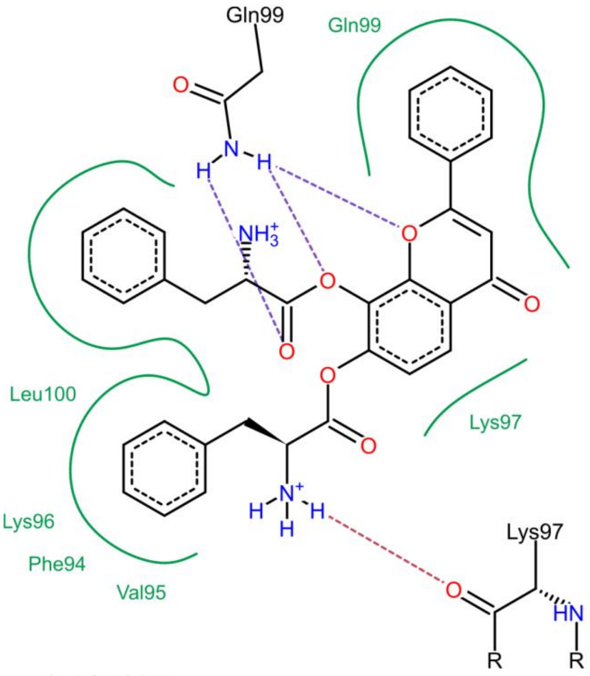

4s (-3.5329)

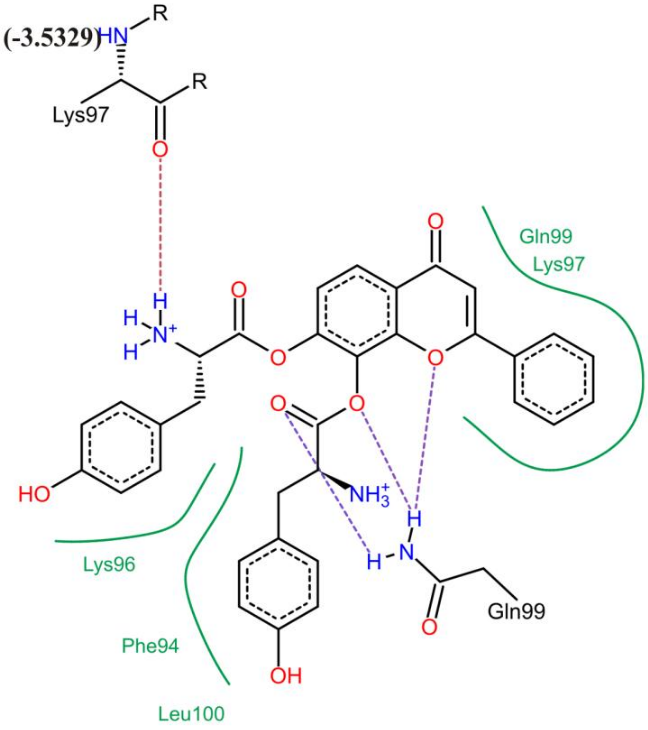

4t (-2.7817)

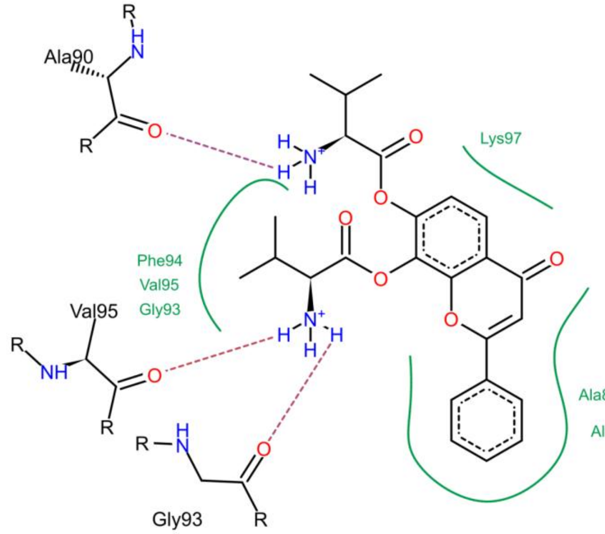

4q (-2.6933)

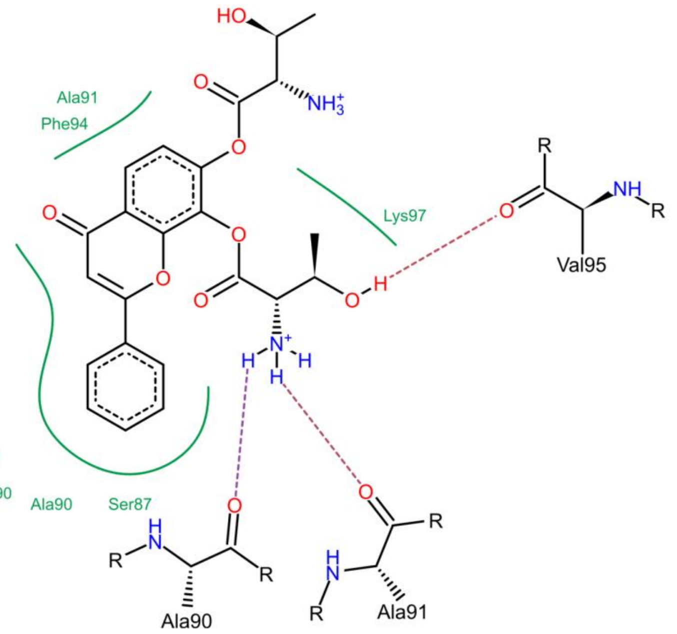

4e (-2.0457)

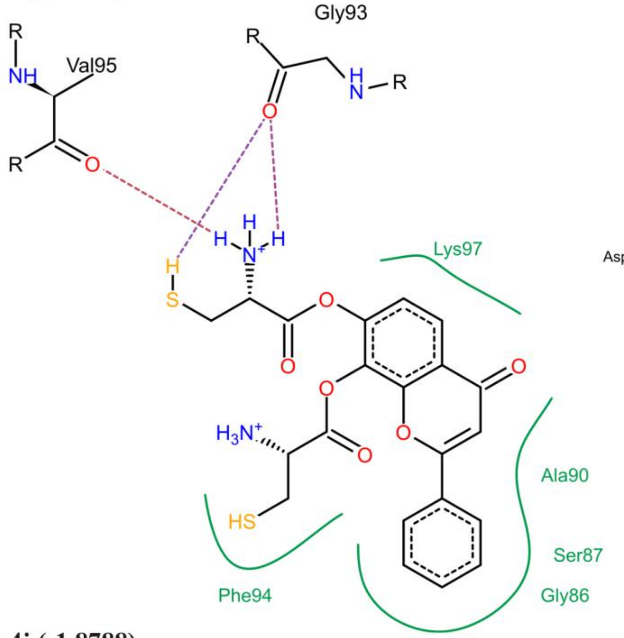

4l (-1.8840)

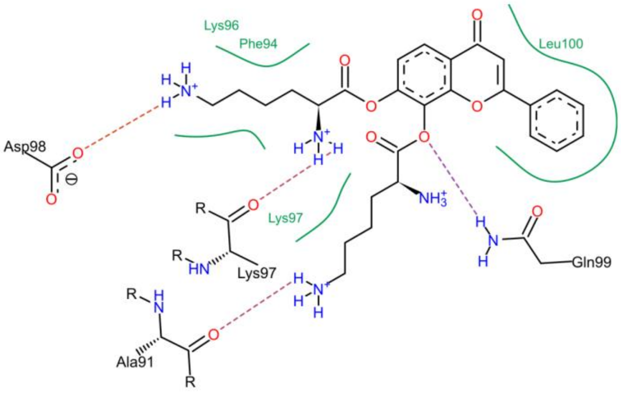

4j (-1.8788)

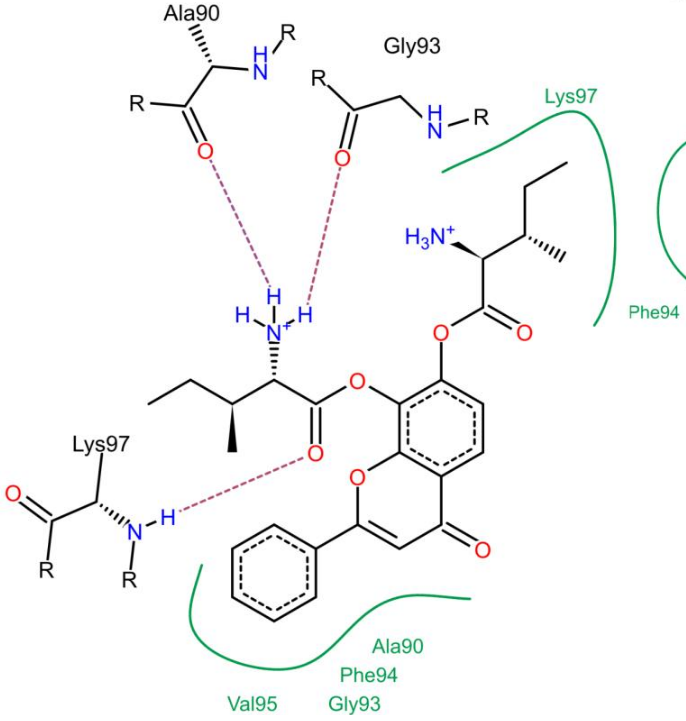

4d (-1.8060)

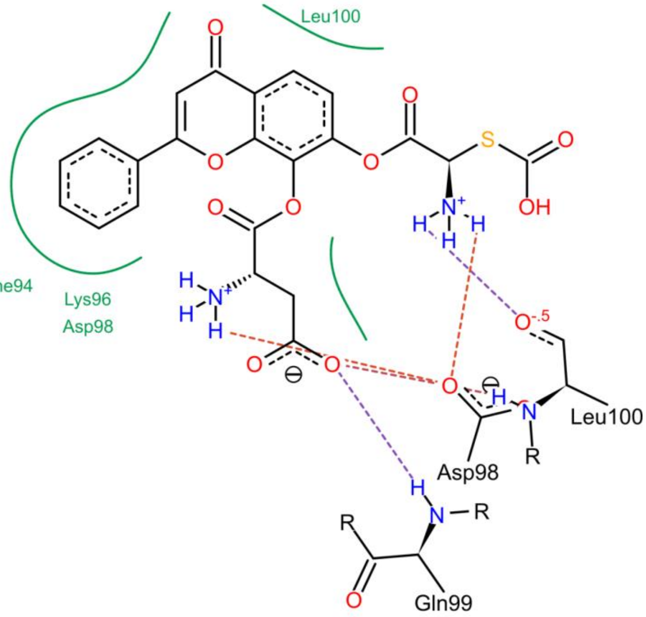

4h (-0.7920)

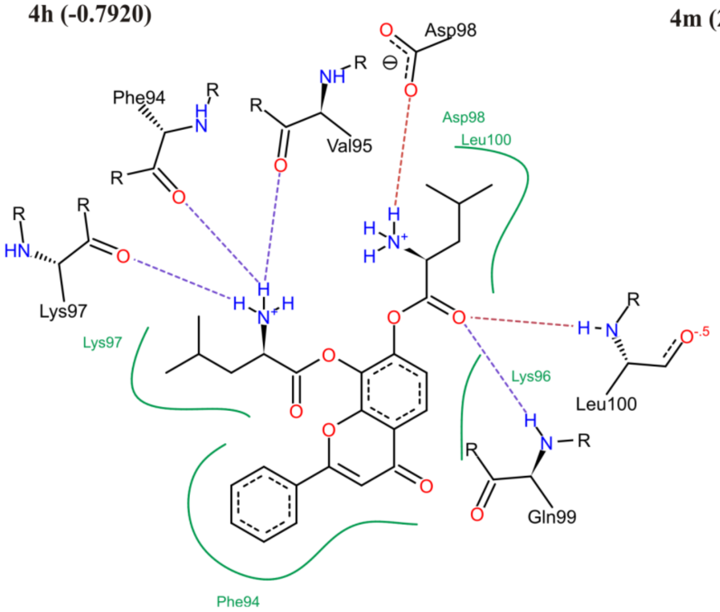

4m (2.3771)

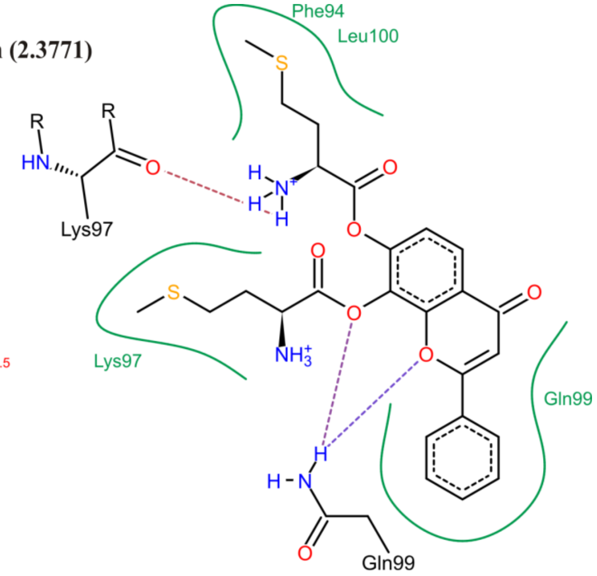

4g (2.7511)

4k (Rejected)

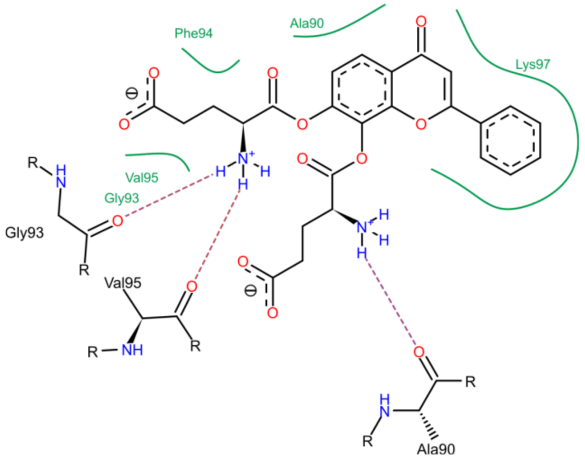

DHF (-14.0383)

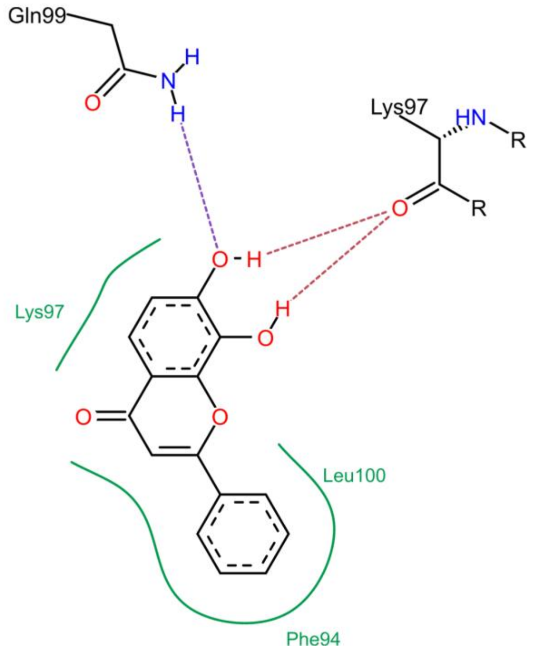

L-DOPA (-9.1560)

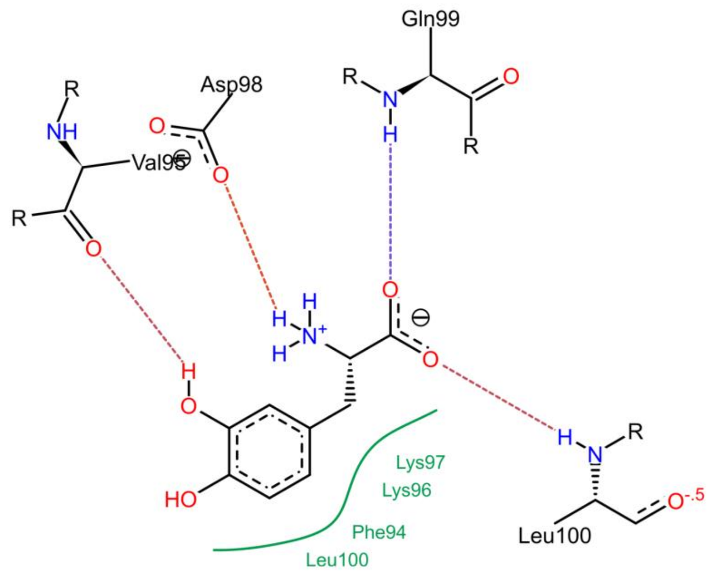

Supplementary Fig. S1. The resulted in docked score (kcal/mol) values on DHF derivatives (**8q-4k**), DHF and L-DOPA molecules with ASN. Compounds and score values (in the bracket) are mentioned in the respective figures.

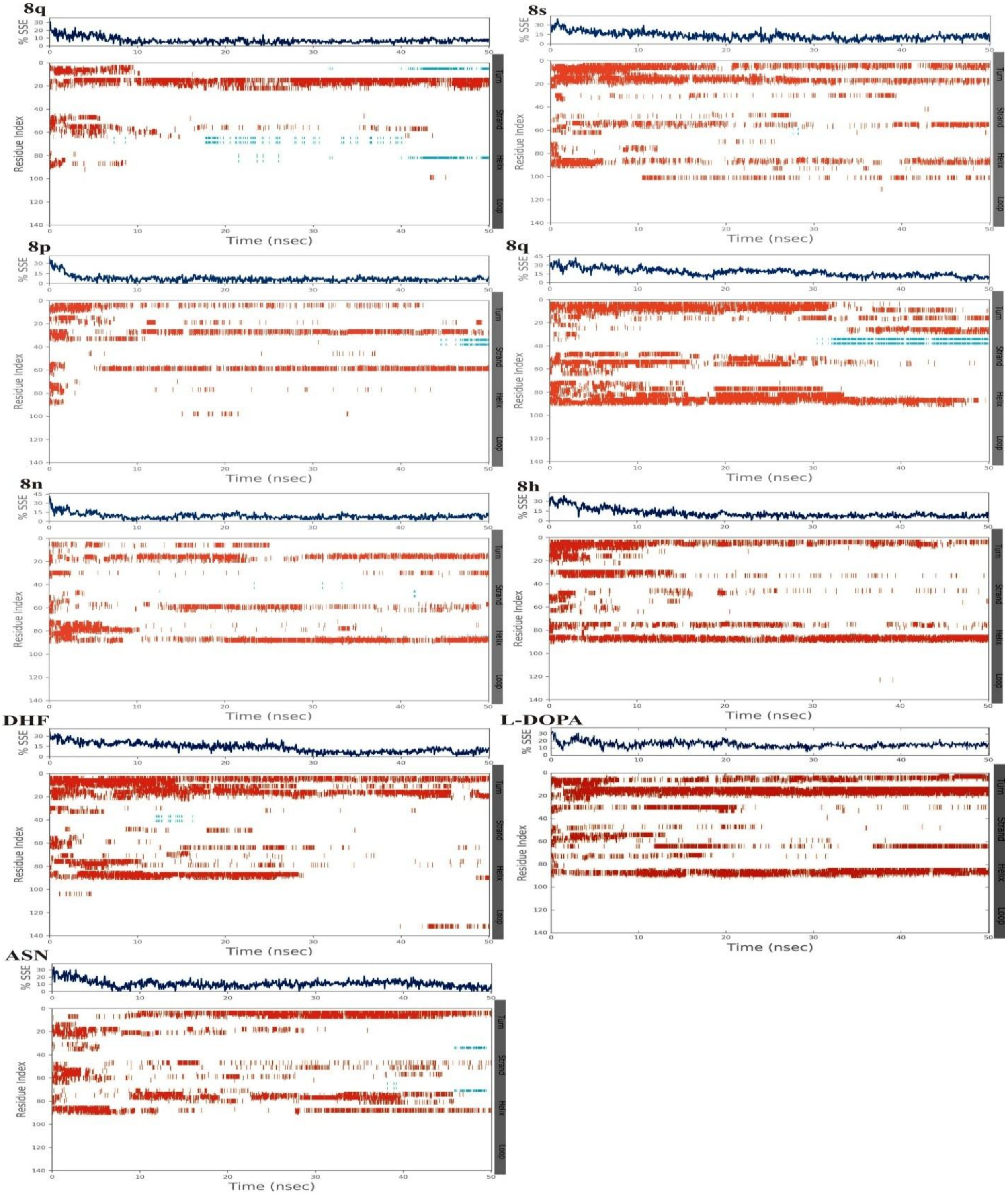

Supplementary Fig. S2. Secondary structure elements (SSE) modification of **8q, 8s, 8p, 8c, 8n, 8h,** DHF and L-DOPA complexes, and ASN alone throughout MD simulation.

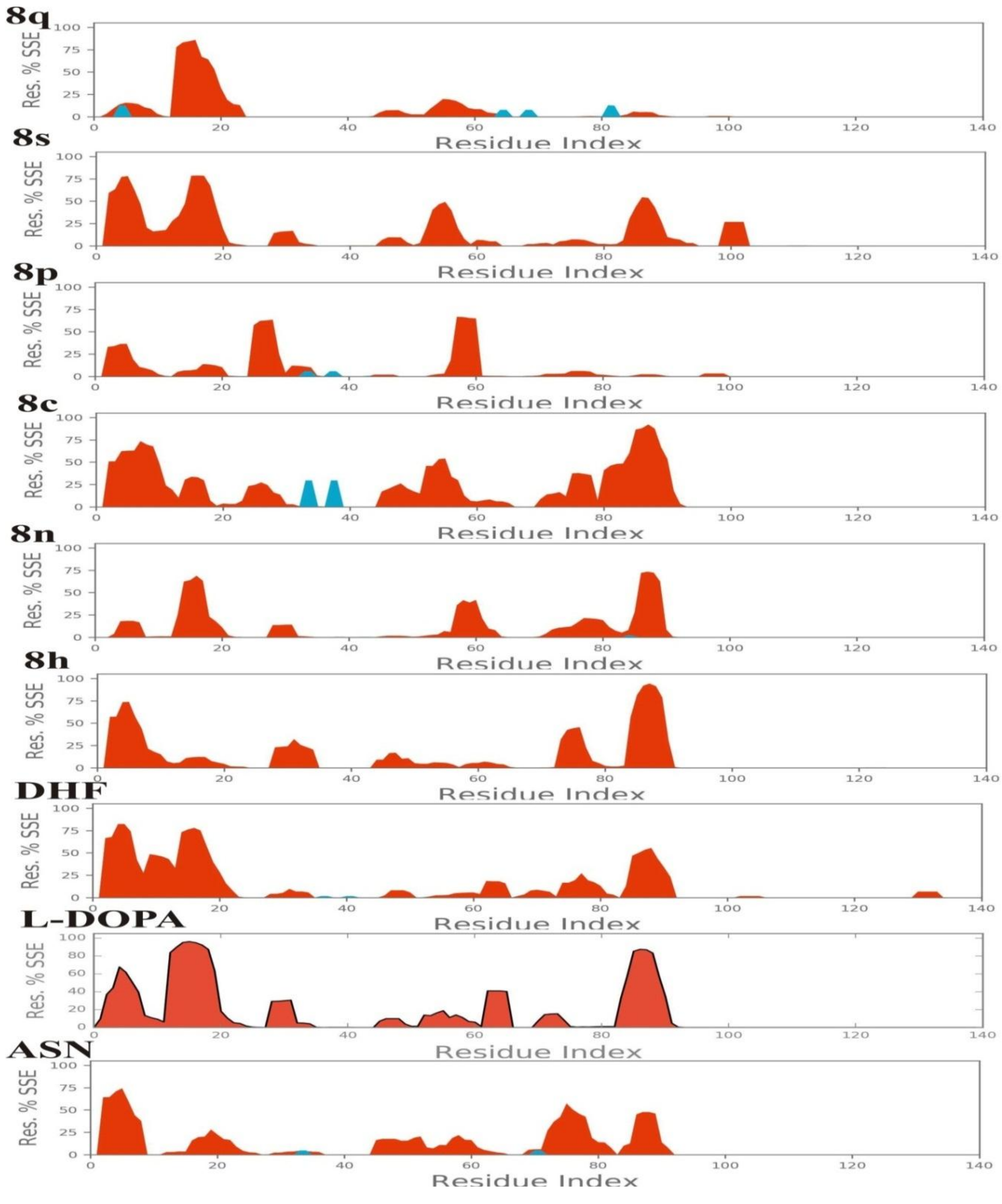

Supplementary Fig. S3. Secondary structure elements (SSE) [alpha-helices (Red) and beta-strands (Blue)] plotted for ASN with **8q, 8s, 8p, 8c, 8n, 8h,** DHF, L-DOPA and ASN alone.

8q

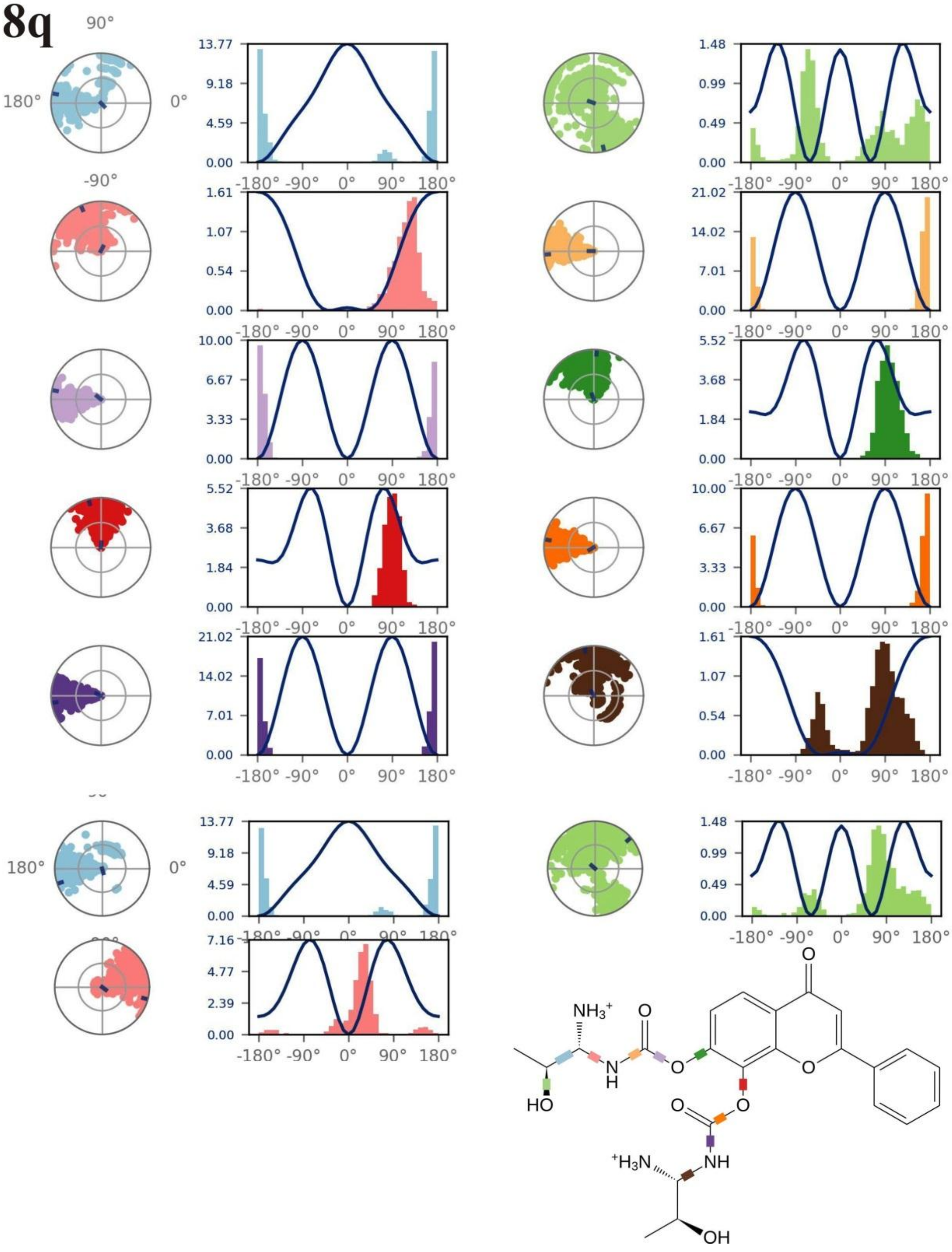

8s

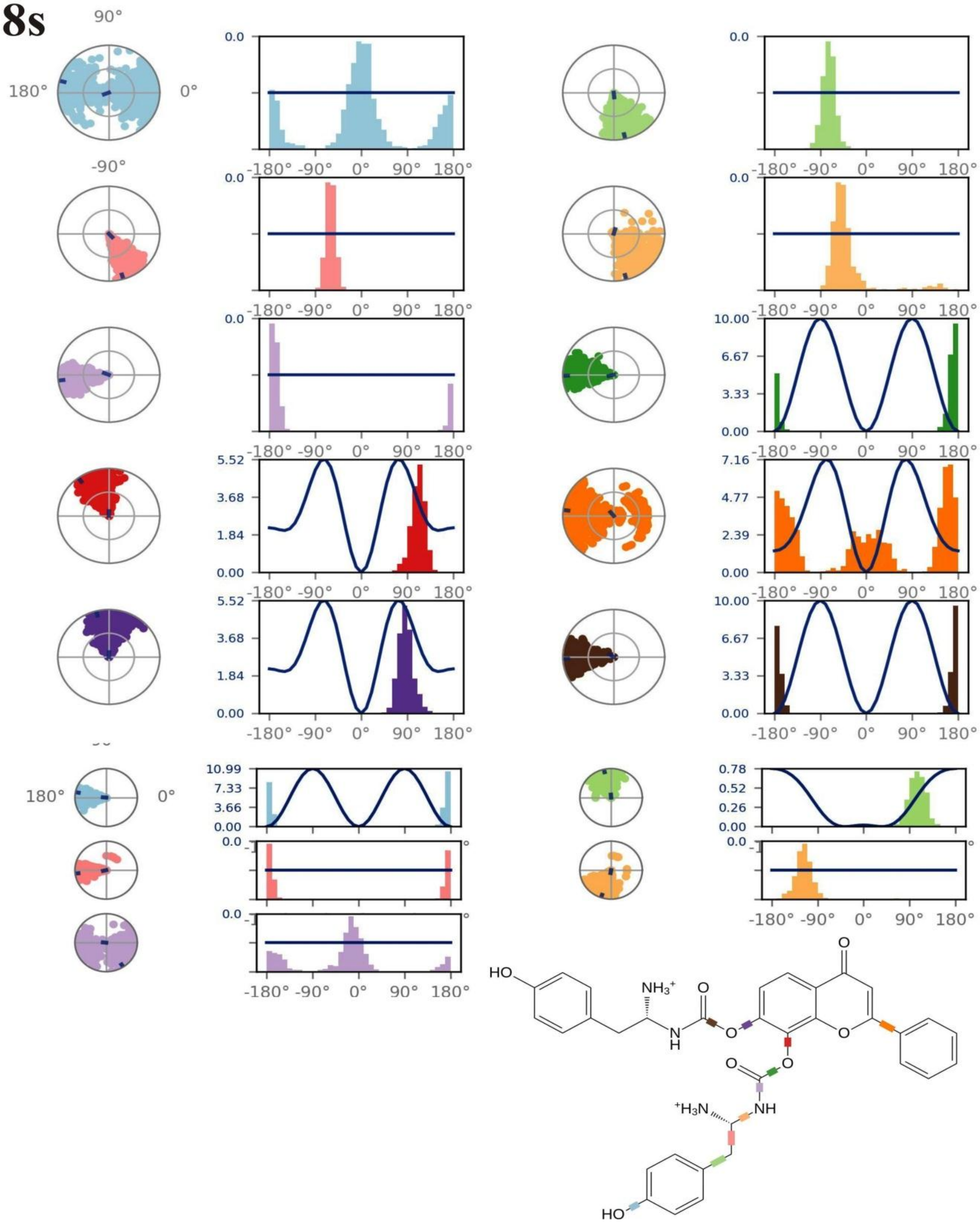

8p

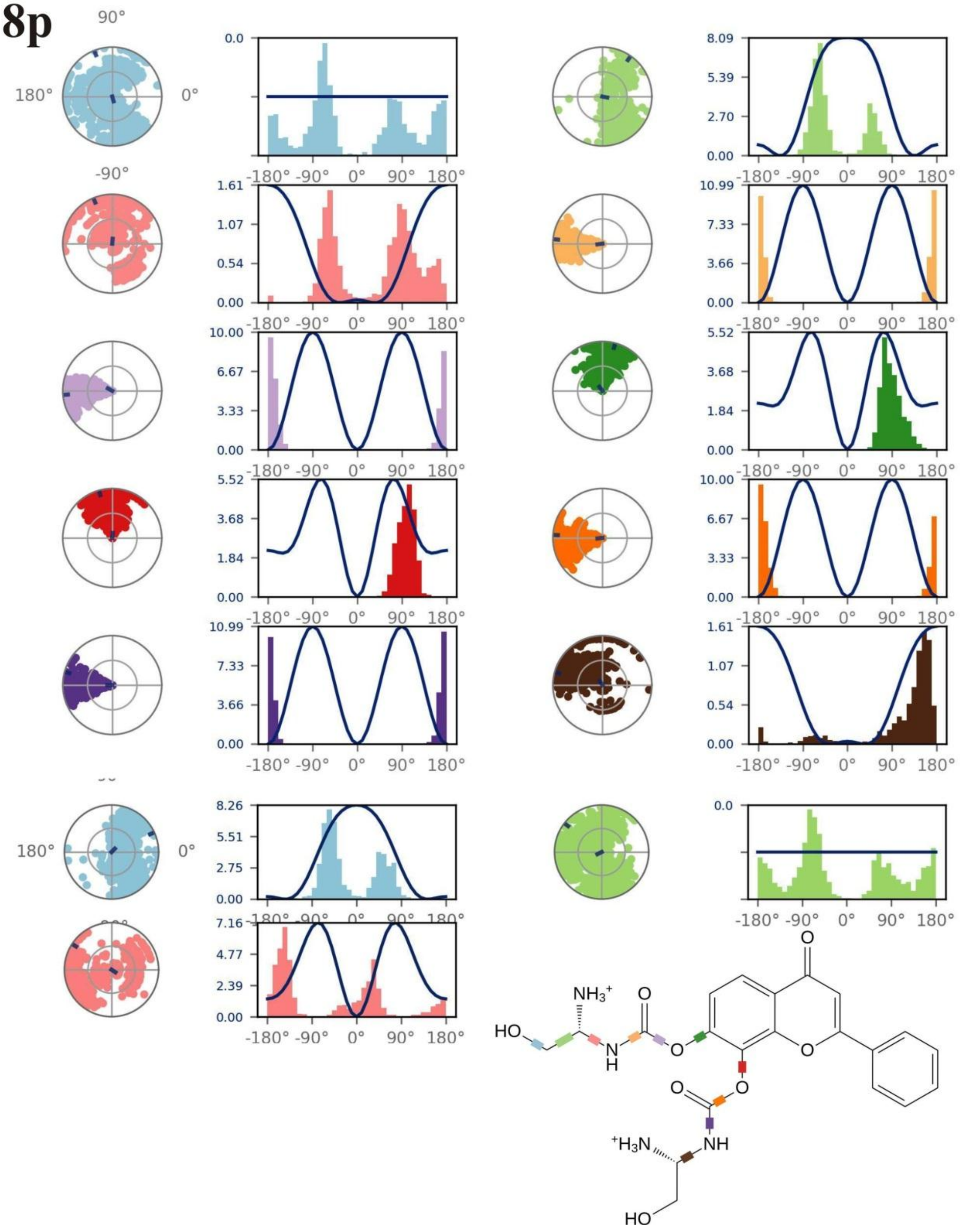

8c

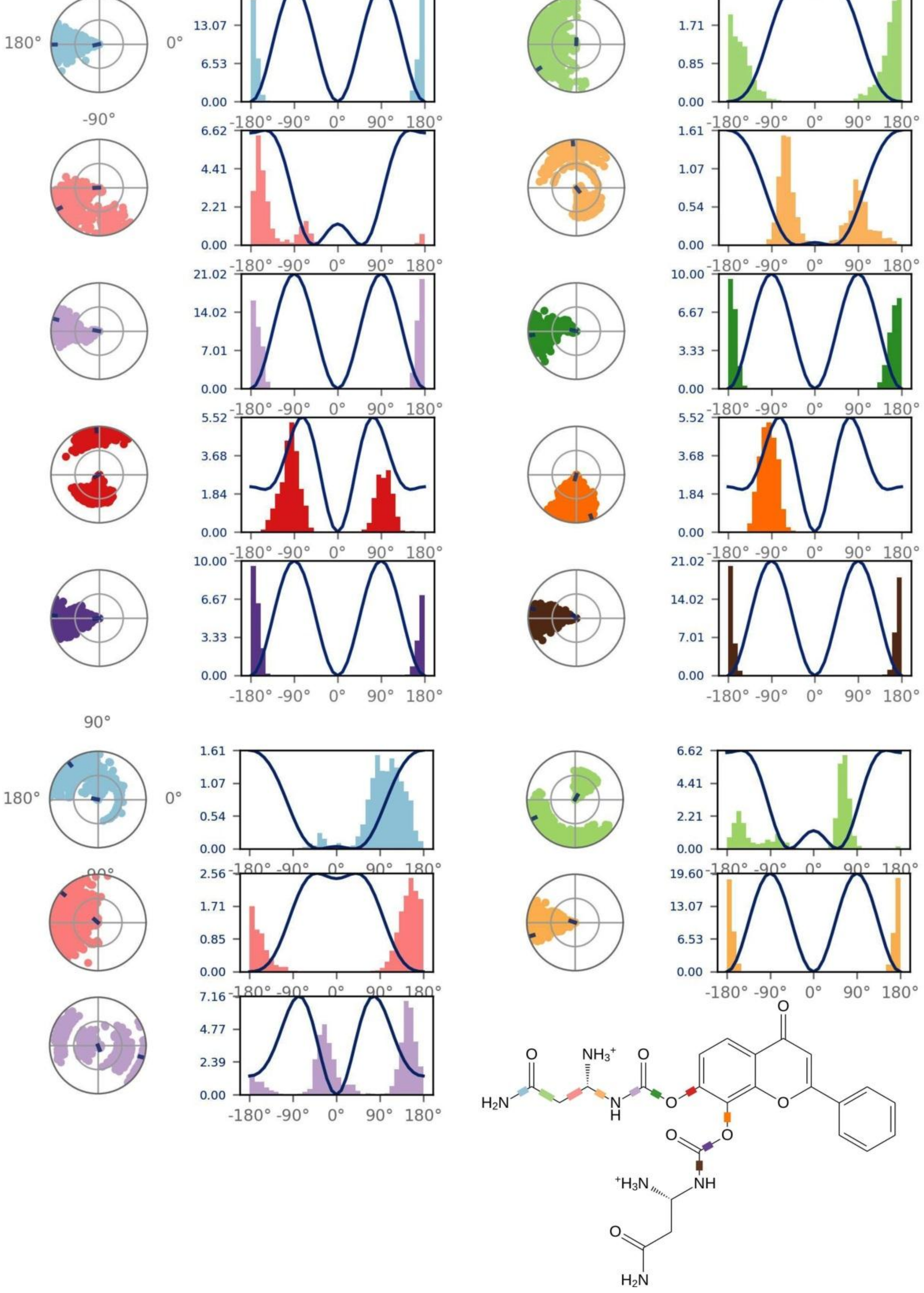

8n

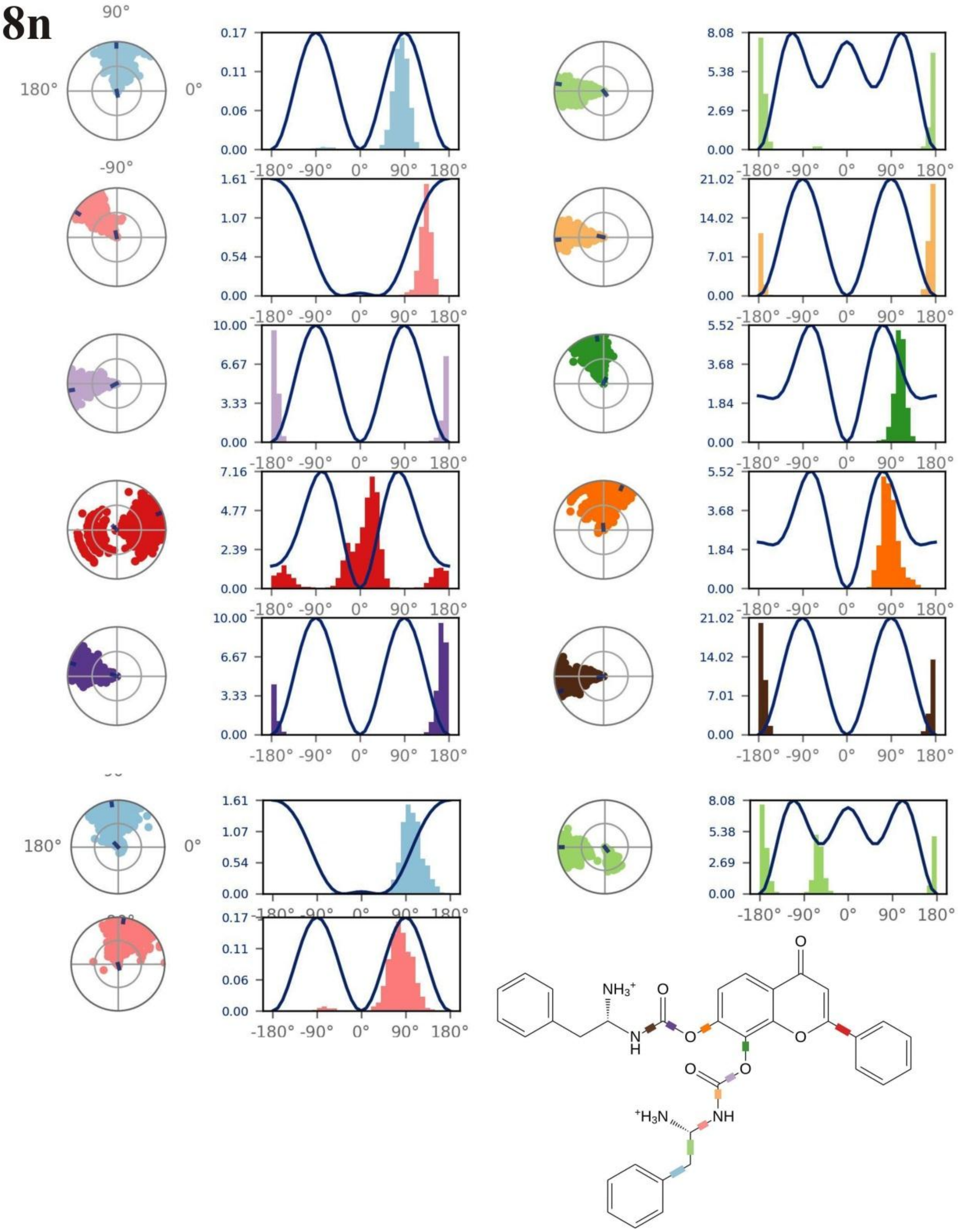

8h

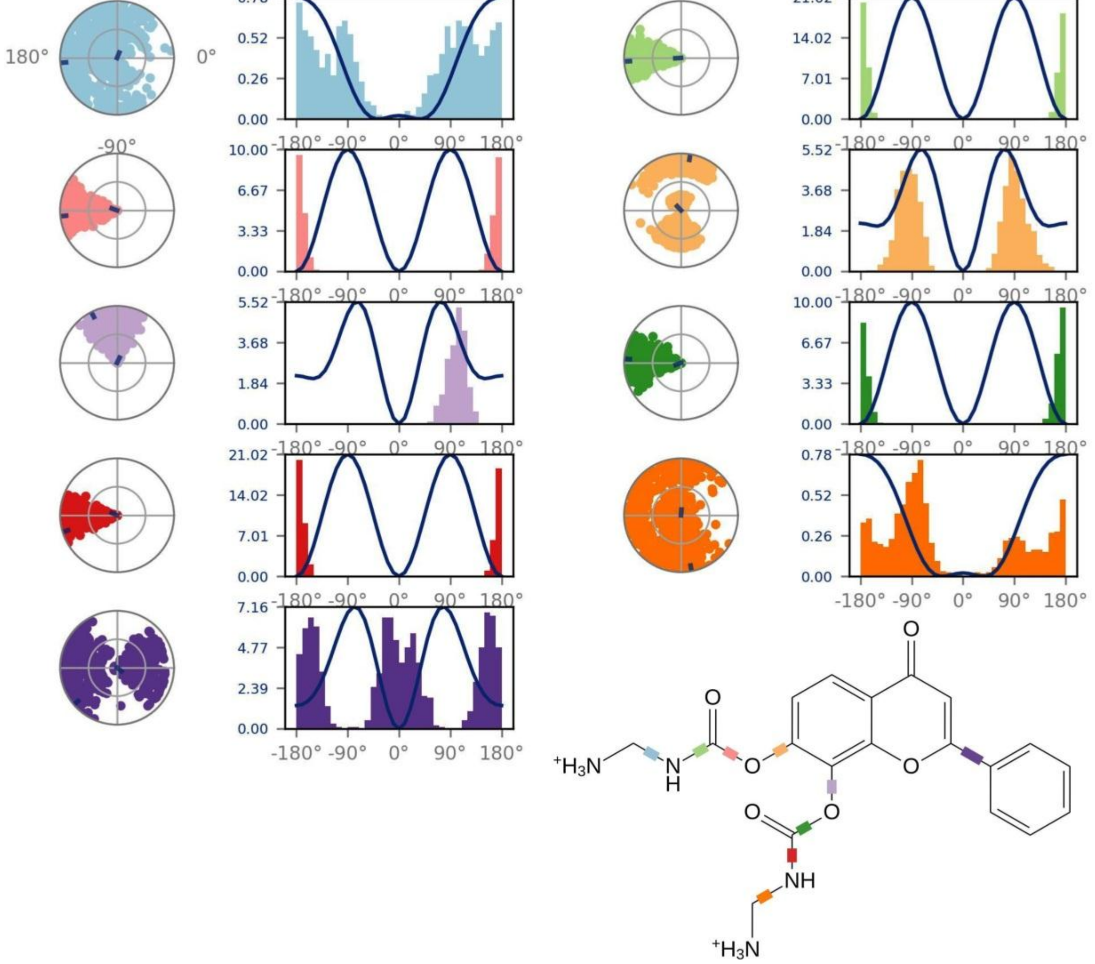
90°
180°
0°
-90°
-180° -90° 0° 90° 180°
O
$^{+}H_3N$
N
H
O
O
O
O
O
NH
$^{+}H_3N$

DHF

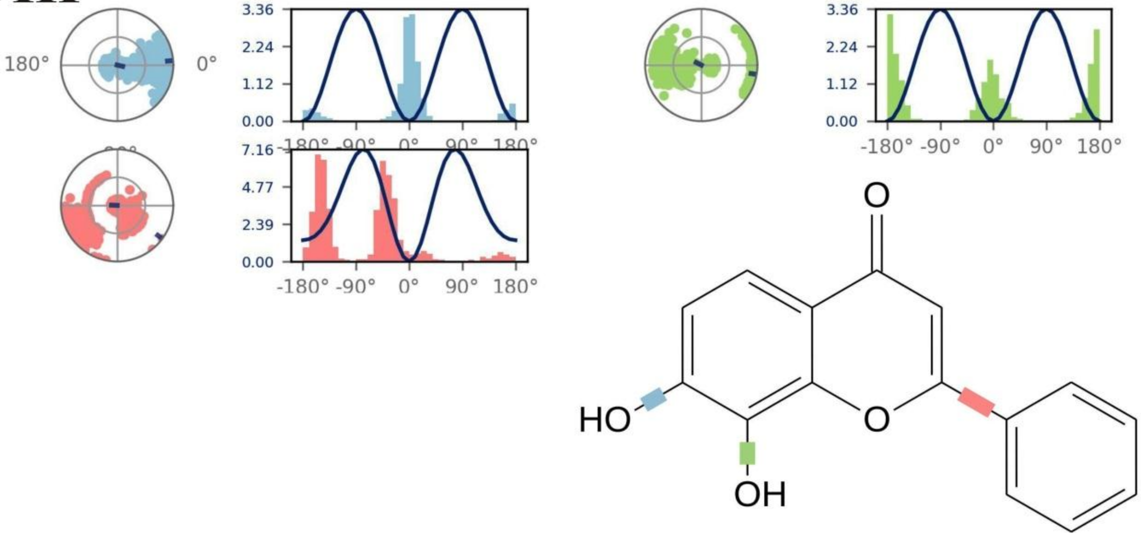
180°
0°
-180° -90° 0° 90° 180°
O
HO
O
OH

L-DOPA

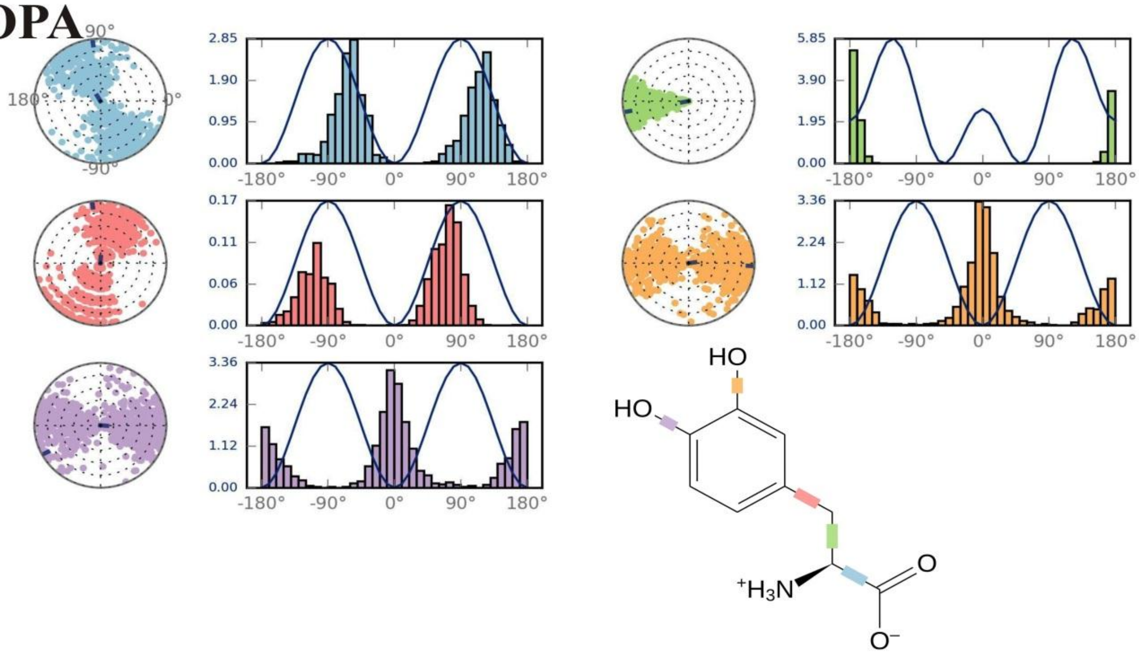

Supplementary Fig. S4. The 2-D circle diagram shows torsion angle of **8q, 8s, 8p, 8c, 8n, 8h,** DHF and L-DOPA.

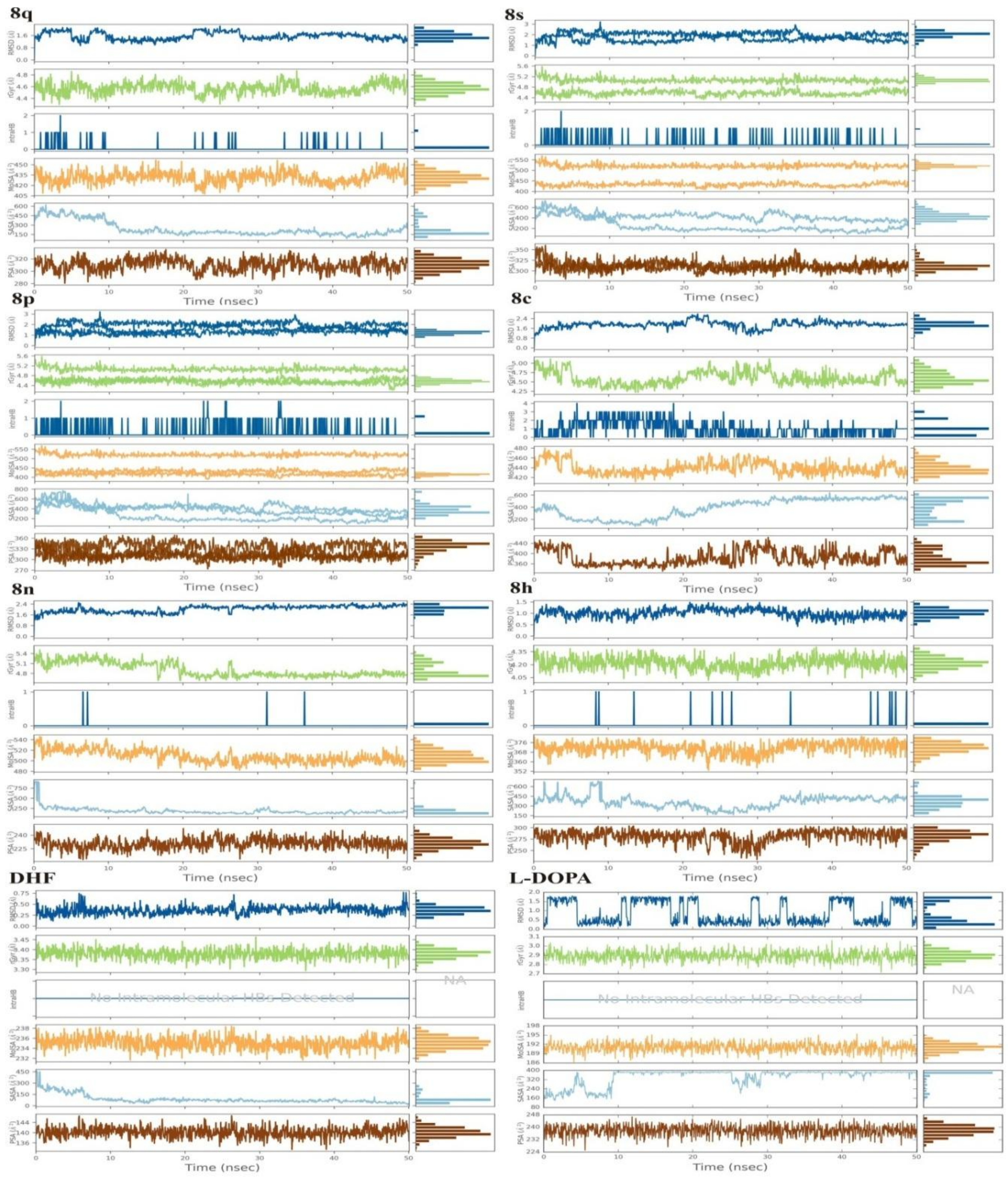

Supplementary Fig. S5. Shows the Ligand RMSD, Radius of Gyration (rGyr), Intramolecular Hydrogen Bonds, Molecular Surface Area (MolSA), Solvent Accessible Surface Area (SASA), Polar Surface Area (PSA) of **8q, 8s, 8p, 8c, 8n, 8h,** DHF, L-DOPA complexes and ASN alone.

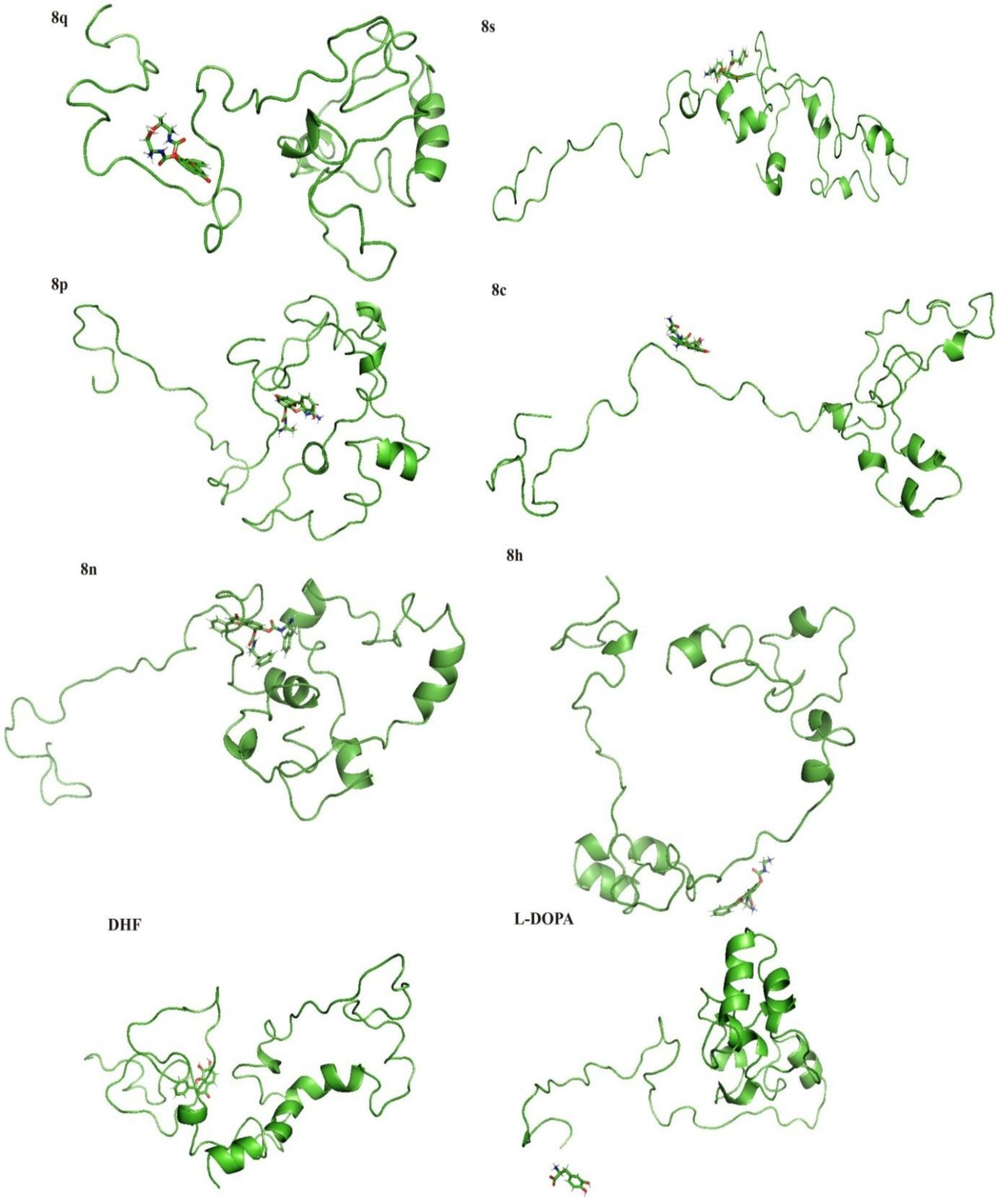

Supplementary Fig. S6. 2D Intermolecular interactions of **8q, 8s, 8p, 8c, 8n, 8h,** DHF and L-DOPA ligands with ASN protein.

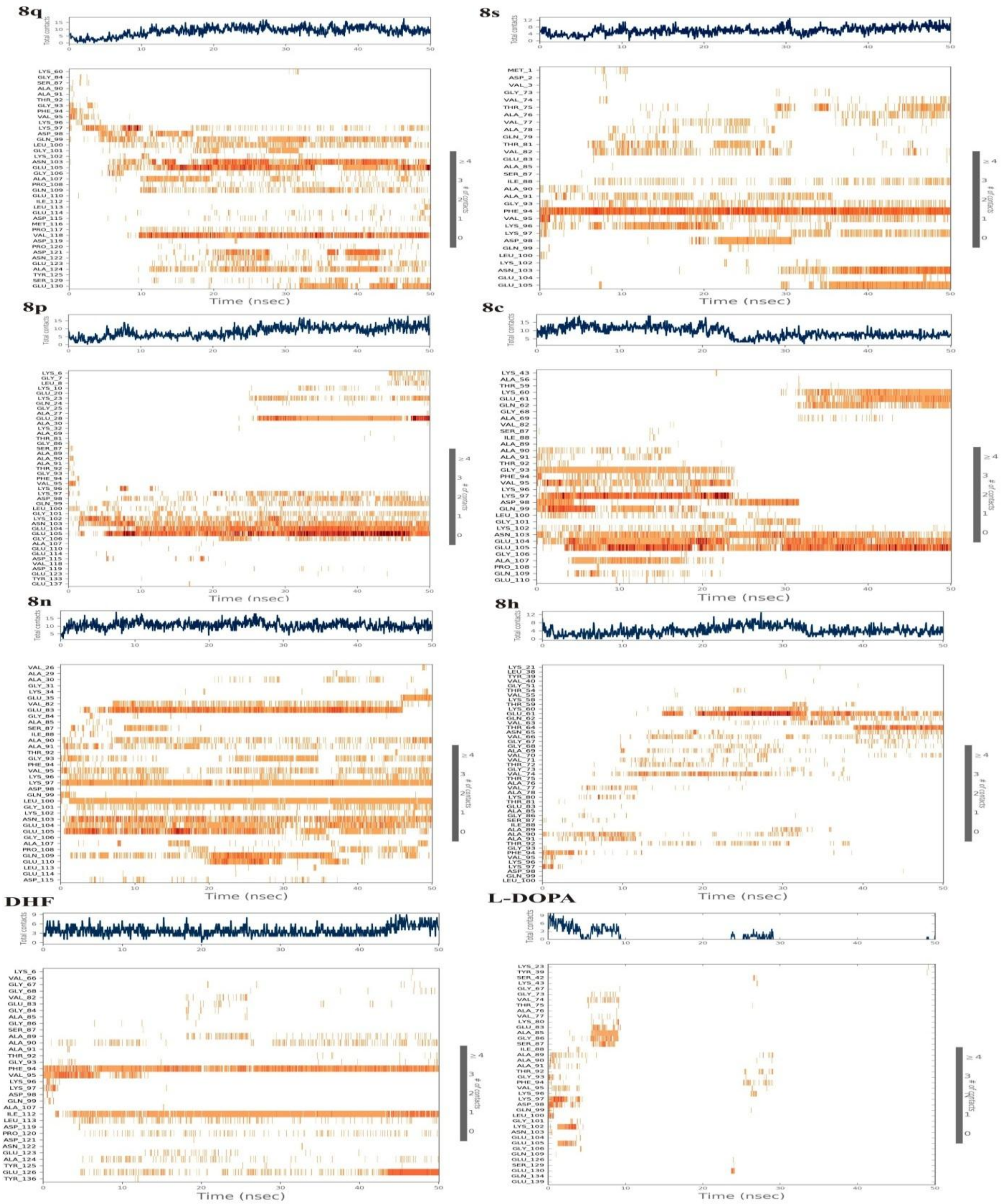

Supplementary Fig. S7. The timeline representation of the interactions between ASN with **8q, 8s, 8p, 8c, 8n, 8h,** DHF and L-DOPA.

Supplementary tables

Supplementary Table S1. The DHF derivative molecules derived from scheme 1 and 2, and used as control (C1, C2) molecules.

**Scheme 1**					
**S No**	**Compound Name**	**Molecular Weight (g/mol)**	**Molecular formula**	**IUPAC Name**	**Chemical structure**
1	DHF-BAP	396.39	$C_{21}H_{20}N_2O_6$	4-oxo-2-phenyl-4*H*-chromene-7,8-diyl bis(2-aminopropanoate	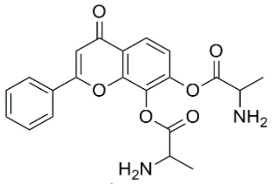 4a
2	DHF-BAGP	566.61	$C_{27}H_{34}N_8O_6$	4-oxo-2-phenyl-4*H*-chromene-7,8-diylbis(2-amino-5-guanidinopentanoate)	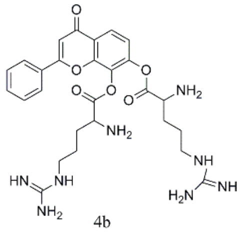 4b
3	DHF-BDOB	482.44	$C_{23}H_{22}N_4O_8$	4-oxo-2-phenyl-4*H*-chromene-7,8-diyl bis(2,4-diamino-4-oxobutanoate)	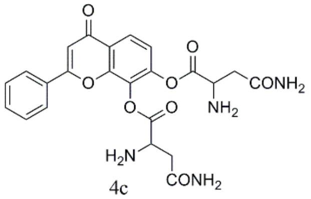 4c
4	DHF-BOBAOA	484.41	$C_{23}H_{20}N_2O_{10}$	4,4'-((4-oxo-2-phenyl-4H-chromene-7,8-diyl)bis(oxy))bis(3-amino-4-oxobutanoic acid)	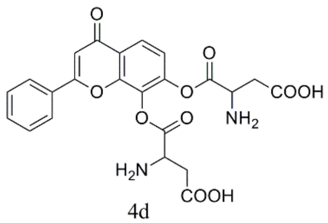 4d
5	DHF-BAMEC	490.55	$C_{21}H_{22}N_4O_6S_2$	4-oxo-2-phenyl-4*H*-chromene-7,8-diylbis(1-amino-2-mercaptoethyl)carbamate)	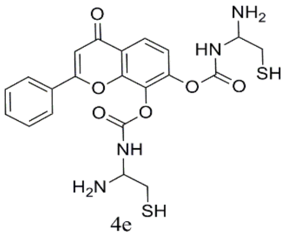 4e

6	DHF-BDOP	510.50	$C_{25}H_{26}N_4O_8$	4-oxo-2-phenyl-4*H*-chromene-7,8-diyl bis(2,5-diamino-5-oxopentanoate)	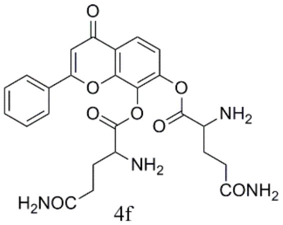 4f
7	DHF-BOBAOPA	512.47	$C_{25}H_{24}N_2O_{10}$	5,5'-((4-oxo-2-phenyl-4*H*-chromene-7,8-diyl)bis (oxo))bis(4-amino-5-oxopentanoic acid)	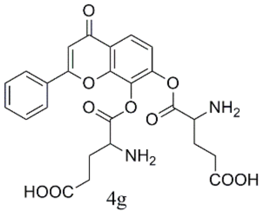 4g
8	DHF-BAMP	480.55	$C_{27}H_{32}N_2O_6$	4-oxo-2-phenyl-4*H*-chromene-7,8-diyl bis(2-amino-4-methylpentanoate)	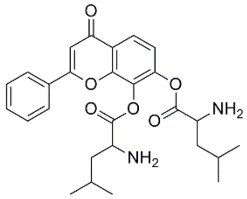 4h
9	DHF-BAIP	528.52	$C_{27}H_{24}N_6O_6$	4-oxo-2-phenyl-4*H*-chromene-7,8-diyl bis(2-amino-3-(1*H*-imidazol-4-yl)propanoate)	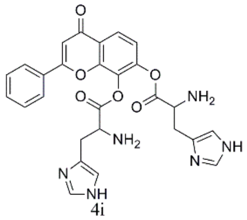 4i
10	DHF-BAMP	480.55	$C_{27}H_{32}N_2O_6$	4-oxo-2-phenyl-4*H*-chromene-7, 8-diyl bis(2-amino-3-methylpentanoate)	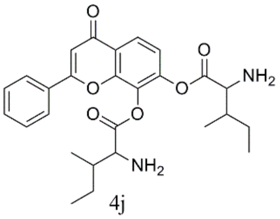 4j
11	DHF-BAMP	480.55	$C_{27}H_{32}N_2O_6$	4-oxo-2-phenyl-4*H*-chromene-7, 8-diyl bis(2-amino-4-methylpentanoate)	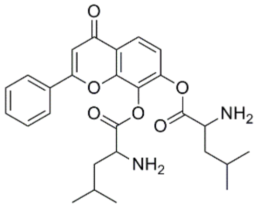 4k

12	DHF-BDH	510.58	$C_{27}H_{34}N_4O_6$	4-oxo-2-phenyl-4*H*-chromene-7,8-diyl bis(2,6-diaminohexanoate)	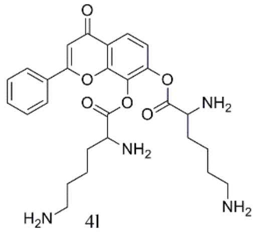 4l
13	DHF-BAMTPC	546.66	$C_{25}H_{30}N_4O_6S_2$	4-oxo-2-phenyl-4*H*-chromene-7,8-diyl bis((1-amino-3-(methylthio)propyl)carbamate)	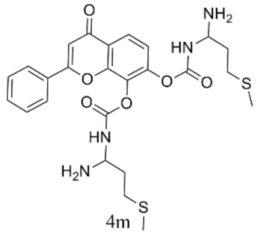 4m
14	DHF-BAPP	548.59	$C_{33}H_{28}N_2O_6$	4-oxo-2-phenyl-4*H*-chromene-7,8-diyl bis(2-amino-3-phenylpropanoate)	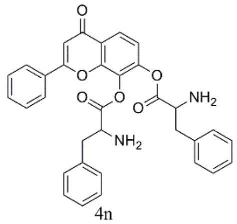 4n
15	DHF-BPC	448.47	$C_{25}H_{24}N_2O_6$	4-oxo-2-phenyl-4*H*-chromene-7,8-diyl bis(pyrrolidine-2-carboxylate	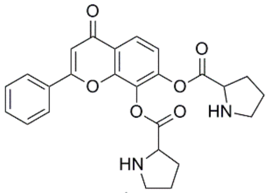 4o
16	DHF-BAHP	428.39	$C_{21}H_{20}N_2O_8$	4-oxo-2-phenyl-4H-chromene-7,8-diyl bis(2-amino-3-hydroxypropanoate)	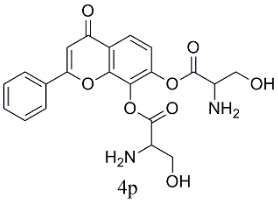 4p
17	DHF-BAHB	456.45	$C_{23}H_{24}N_2O_8$	4-oxo-2-phenyl-4*H*-chromene-7,8-diyl bis(2-amino-3-hydroxybutanoate)	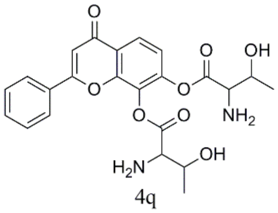 4q

18	DHF-BAIP	626.66	$C_{37}H_{30}N_4O_6$	4-oxo-2-phenyl-4*H*-chromene-7, 8-diyl bis(2-amino-3 -(1*H*-indol-3-yl)propanoate)	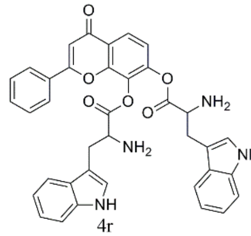 4r
19	DHF-BAHPP	580.58	$C_{33}H_{28}N_2O_8$	4-oxo-2-phenyl-4*H*-chromene-7,8-diyl bis(2-amino-3-(4-hydroxyphenyl)propanoate)	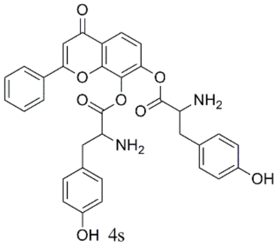 4s
20	DHF-BAMB	452.50	$C_{25}H_{28}N_2O_6$	4-oxo-2-phenyl-4*H*-chromene-7, 8-diyl bis(2-amino-3-methylbutanoate)	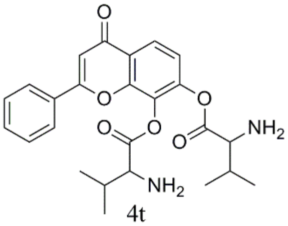 4t
**Scheme 2**					
21	DHF-ACOOPCAAA	440.45	$C_{22}H_{24}N_4O_6$	7-(((1-aminoethyl) carbamoyl)oxy)-4-oxo-2-phenyl-4*H*-chromen-8-yl 2-((1-aminoethyl)amino)acetate	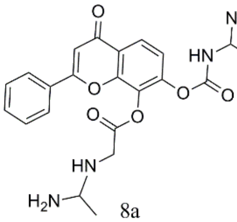 8a
22	DHF-BAGBC	596.64	$C_{27}H_{36}N_{10}O_6$	4-oxo-2-phenyl-4*H*-chromene-7,8-diyl bis((1-amino-4-guanidinobutyl)carbamate)	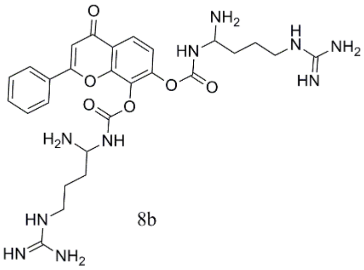 8b

23	DHF-BDOPC	512.47	$C_{23}H_{24}N_6O_8$	4-oxo-2-phenyl-4*H*-chromene-7,8-diyl bis((1, 3-diamino-3 -oxopropyl)carbamate)	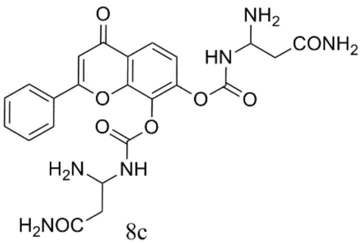 8c
24	DHF-BOBCBABAPA	514.44	$C_{22}H_{23}N_4O_{10}$	3,3’-((((4-oxo-2-phenyl-4*H*-chromene-7,8-diyl)bis(oxy))bis (carbonyl))bis(azanediyl)) bis(3-aminopropanic acid)	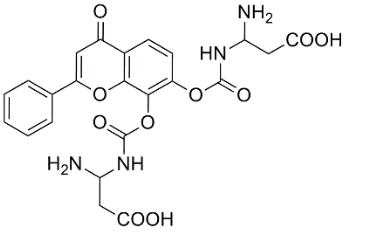 8d
25	DHF-BAMEC	490.55	$C_{21}H_{22}N_4O_6S_2$	4-oxo-2-phenyl-4*H*-chromene-7,8-diyl bis((1-amino-2 mercaptoethyl)carbamate)	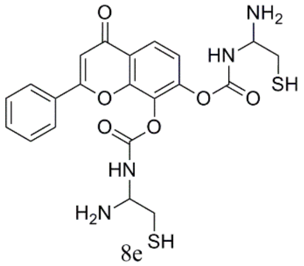 8e
26	DHF-BDOBC	540.53	$C_{25}H_{28}N_6O_8$	4-oxo-2-phenyl-4*H*-chromene-7,8-diyl bis((1,4-diamino-4-oxobutyl)carbamate	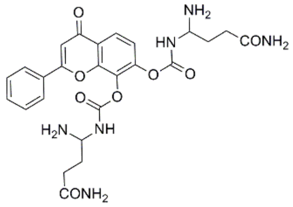 8f
27	DHF-BOBCBABABA	542.49	$C_{25}H_{26}N_4O_{10}$	4,4’-((((4-oxo-2-phenyl-4*H*-chromene-7,8-diyl)bis(oxy))bis (carbonyl))bis(azenediyl)) bis(4-aminobutanoic acid)	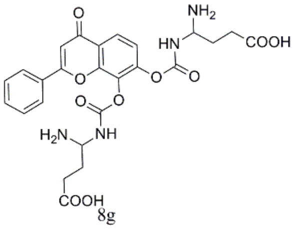 8g
28	DHF-BAMC	398.37	$C_{19}H_{18}N_4O_6$	4-oxo-2-phenyl-4*H*-chromene-7,8-diyl bis((aminomethyl) carbamate)	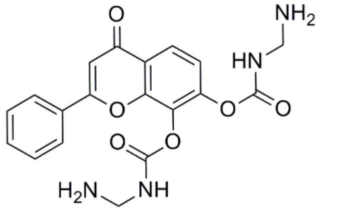 8h

29	DHF-BAIEC	558.55	$C_{27}H_{26}N_8O_6$	4-oxo-2-phenyl-4*H* -chromene-7,8-diyl bis((1-amino-2-(1*H*-imidazol-4-yl) ethyl)carbamate)	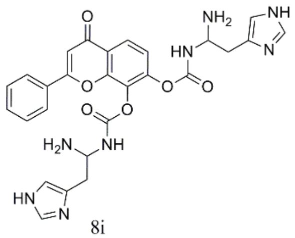 8i
30	DHF-BAMBC	510.58	$C_{27}H_{34}N_4O_6$	4-oxo-2-phenyl-4*H*-chromene-7,8-diyl bis((1-amino-2-methylbutyl)carbamate	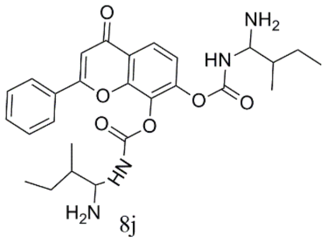 8j
31	DHF-AMCOOPCAMAA	524.61	$C_{28}H_{36}N_4O_6$	7-(((1-amino-3 -methylbutyl)carbamoyl) oxy)-4-oxo-2-phenyl-4*H*-chromen-8-yl 2-((1-amino-3-methylbutyl)amino) acetate	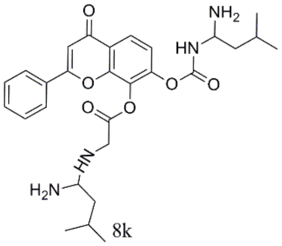 8k
32	DHF-BDPC	540.61	$C_{27}H_{36}N_6O_6$	4-oxo-2-phenyl-4*H*-chromene-7,8-diyl bis((1,5-diaminopentyl) carbamate)	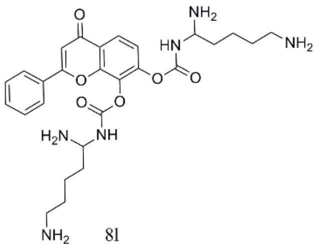 8l
33	DHF-BAMTPC	546.66	$C_{25}H_{30}N_4O_6S_2$	4-oxo-2-phenyl-4*H*-chromene-7,8-diyl bis(2-amino-3-(methylthio) propyl)carbamate	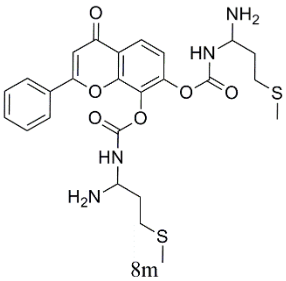 8m

34	DHF-BAPEC	578.61	$C_{33}H_{30}N_4O_6$	4-oxo-2-phenyl-4*H*-chromene-7,8-diyl bis ((1-amino-2-phenylethyl) carbamate)	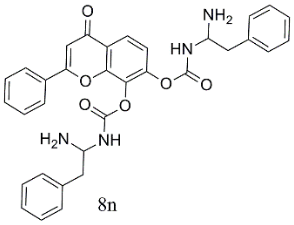 8n
35	DHF-BOBCBABPCA	566.52	$C_{27}H_{26}N_4O_{10}$	1,1’-((((4-oxo-2-phenyl-4*H*-chromene-7,8-diyl)bis(oxy))bis(carbonyl)) bis(azanediyl))bis (pyrrolidine-2-carboxylic acid)	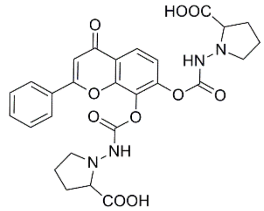 8o
36	DHF-BAHEC	458.42	$C_{21}H_{22}N_4O_8$	4-oxo-2-phenyl-4H-chromene-7,8-diyl bis((1-amino-2-hydroxyethyl) carbamate)	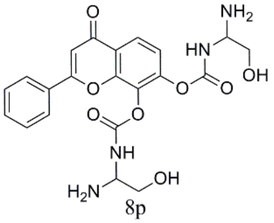 8p
37	DHF-BAHPC	486.47	$C_{23}H_{26}N_4O_8$	4-oxo-2-phenyl-4*H*-chromene-7,8-diyl bis((1-amino-2-hydroxypropyl) carbamate)	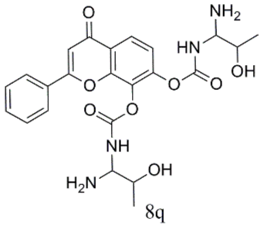 8q
38	DHF-BAIEC	656.69	$C_{37}H_{32}N_6O_6$	4-oxo-2-phenyl-4*H*-chromene-7,8-diyl bis((1-amino-2-(1*H*-indol-3-yl) ethyl)carbamate	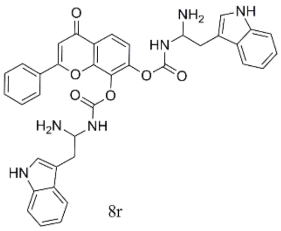 8r
39	DHF-BAHPEC	610.61	$C_{33}H_{30}N_4O_8$	4-oxo-2-phenyl-4*H*-chromene-7,8-diyl bis((1-amino-2-(4-hydroxyphenyl) ethyl)carbamate)	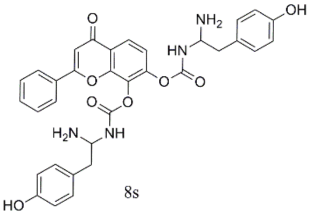 8s

40	DHF-AMOPCAMAA	496.56	$C_{26}H_{32}N_4O_6$	7-(((1-amino-2-methylpropyl)oxy)-4-oxo-2-phenyl-4*H*-chromen-8-yl 2-((1-amino-2-methylpropyl)amino)acetate	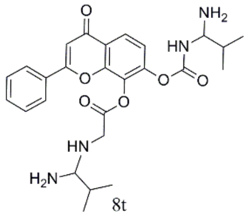 8t
**Pubmed**					
C1	DHF	254.23	$C_{15}H_{10}O_4$	7,8-dihydroxy-2-phenyl-4*H*-chromen-4-one	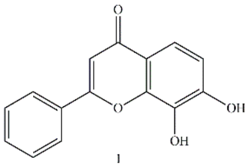 1
C2	L-DOPA	197.18	$C_9H_{11}NO_4$	(s)-2-amino-3-(3,4-dihydroxyphenyl) propanoic acid	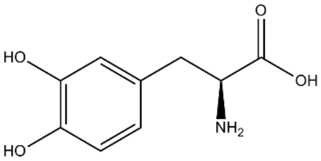

Supplementary Table S2. The intermolecular interaction of lead compounds **8q, 8s, 8p, 8c, 8n, 8h,** DHF and L-DOPA with active site amino acids of ASN.

**Compound**	**LeadIT (Docking)**					
	**LeadIT score**	**H-bond**	**Amino acid**	**Amino acid atom**	**Ligand atom**	**H-bond Length (Å)**
8q	-16.3120	7	Gln99	HE22	O17	1.9
			Ala90	O	H54	2.0
			Gly93	O	H55	2.3
			Ser87	O	H58	2.0
			Val95	O	H60	2.0
			Ala90	O	H61	2.1
			Gly93	O	H62	1.8
			Gly93	O	H47	2.7
			Lys97	Pi-Cation		4.7
			Lys96	Pi-Alkyl		5.4
			Leu100			4.9
			Lys97			4.6
			Lys97			5.1
8s	-16.1875	7	Lys97	HN	O25	1.9
			Lys96	HA	O25	3.0
			Leu100	HN	O17	2.5
			Gln99	HN	O17	1.9
			Gly93	O	H76	2.1
			Ser87	O	H75	1.9
			Val95	O	H73	1.4
			Val95	Amide-Pi Stacked		4.8
			Lys96	Pi-Sigma		2.1
			Lys97	Pi-Lone Pair		2.6
			Ala90	Pi-Alkyl		4.5
			Ala91			4.3
			Leu100			4.6
			Lys96			5.2
			Lys97			4.2
8p	-15.2223	8	Gln99	HE22	O17	1.9
			Ala90	O	H48	2.0
			Gly93	O	H49	2.3
			Ser87	O	H52	2.0
			Val95	O	H54	2.0
			Ala90	O	H55	2.1
			Gly93	O	H56	1.8

			Gly93	O	H46	2.7
			Lys97	Pi-Cation		4.7
			Lys96	Pi-Alkyl		5.4
			Leu100			4.9
			Lys97			4.6
			Lys97			5.1
8c	-14.3118	9	Gln99	HE22	O17	2.5
			Asp98	OD1	H55	1.4
			Val95	O	H53	1.7
			Val95	O	H57	2.4
			Phe94	O	H58	2.1
			Ala90	O	H60	2.7
			Ala91	O	H60	2.2
			Gly93	O	H62	2.2
			Ala90	HA	O36	3.1
			Lys97	Pi-Cation		4.2
			Lys97	Pi-Sigma		2.4
			Phe94	Pi-Pi T-shaped		5.3
			Ala91	Pi-Alkyl		5.1
			Lys97			4.4
			Leu100			4.9
8n	-14.2893	5	Lys97	HN	O25	1.9
			Gln99	HN	O17	1.9
			Leu100	HN	O17	2.5
			Val95	O	H73	1.5
			Gly93	O	H74	2.0
			Lys96	Pi-Sigma		2.2
			Lys97	Pi-Lone pair		2.5
			Val95	Amide-Pi Stacked		4.7
			Leu100	Pi-Alkyl		4.6
			Lys97			4.2
			Ala90			4.3
			Ala91			4.5
8h	-14.2810	6	Ser87	O	H44	2.0
			Gln99	HE22	O17	1.9
			Ala90	O	H42	2.0
			Ala90	O	H43	2.3
			Val95	O	H46	2.3
			Gly93	O	H47	1.5
			Lys97	Pi-Cation		4.3
			Leu100	Pi-Alkyl		4.2
			Lys97			4.5
DHF	-14.0383	3	Gln99	HE22	O17	2.1
			Lys97	O	H28	1.9
			Lys97	O	H29	2.0

			Phe94	Pi-Pi Stacked		3.8
			Leu100	Pi-Alkyl		4.4
L-DOPA	-9.1560	5	Asp98	OD1	H24	1.6
			Phe94	HA	O1	3.1
			Gln99	HN	O4	2.3
			Leu100	HN	O4	2.2
			Val95	O	H21	1.7
			Phe94	Pi-Sigma		2.2
			Lys97	Attractive Charge		5.3
			Lys97	Pi-Alkyl		5.3
			Leu100			5.3

Supplementary Table S3. The ADMET properties and AMES mutagenicity studies for lead compounds **8q, 8s, 8p, 8c, 8n, 8h** DHF and L-DOPA.

**Compound**	**8q**	**8s**	**8p**	**8c**	**8n**	**8h**	**DHF**	**L-DOPA**
Molecular weight	486.47	610.61	458.42	512.47	578.61	398.37	254.23	197.18
H-bond donor	6	6	6	6	4	4	2	3
H-bond acceptor	8	8	8	8	6	6	4	4
log P	-0.929	2.203	-1.684	-2.401	2.687	-1.04	2.652	-2.089
AQ SOL LEV	3	2	3	2	2	3	3	5
BBB LEV	4	4	4	4	4	4	2	4
CYP 2D6	-7.87546	-4.97894	-7.87546	-9.93245	-6.27104	-8.15065	-5.77583	-6.85987
HEPATOX	-0.436636	-2.55554	-1.2086	-0.111251	-3.39089	-0.945906	-1.28197	-4.01274
PPB LEV	-5.30747	-7.08034	-6.1351	-8.50688	-1.34427	-3.32076	2.10687	-11.929
NTP Carcinogenicity Call (Male Mouse) (v3.2)-TOPKAT	1.000	1.000	1.000	1.000	1.000	1.000	1.000	0.000
NTP Carcinogenicity Call (Female Mouse) (v3.2)-TOPKAT	0.000	0.082	0.000	0.000	0.002	0.000	0.000	0.000
Developmental Toxicity Potential (DTP) (v3.1)-TOPKAT	1.000	1.000	1.000	1.000	1.000	1.000	0.999	0.293
Skin Irritation (v6.1)-TOPKAT	0.000	0.000	0.247	1.000	0.000	0.000	0.000	0.303
Ames Mutagenicity (v3.1)-TOPKAT	0.000	0.000	0.000	0.000	0.000	1.000	0.000	0.000

Solubility: 0-2 highly soluble, **BBB:** 2- medium penetration and 3- low penetration, **CYP2D6:** -ve – non-inhibitors & +ve –inhibition.

HEPATOX: 0-1: Non-toxic, **PPB:** Greater the value greater the binding capacity and toxicity analysis using TOPKAT.

Note: 0- Negative result, **1:** positive results
